# Smallest Secondary Nucleation Competent Aβ Aggregates Probed by an ATP-Independent Molecular Chaperone Domain

**DOI:** 10.1021/acs.biochem.1c00003

**Published:** 2021-02-23

**Authors:** Axel Leppert, Ann Tiiman, Nina Kronqvist, Michael Landreh, Axel Abelein, Vladana Vukojević, Jan Johansson

**Affiliations:** † Department of Neurobiology, Care Sciences and Society, Division of Neurogeriatrics, Karolinska Institutet, 14183 Huddinge, Sweden; ‡ Department of Clinical Neuroscience, Center for Molecular Medicine, Karolinska Institutet, 17176 Stockholm, Sweden; § Department of Microbiology, Tumour and Cell Biology, Karolinska Institutet, Biomedicum, Solnavägen 9, 17165 Solna, Sweden

## Abstract

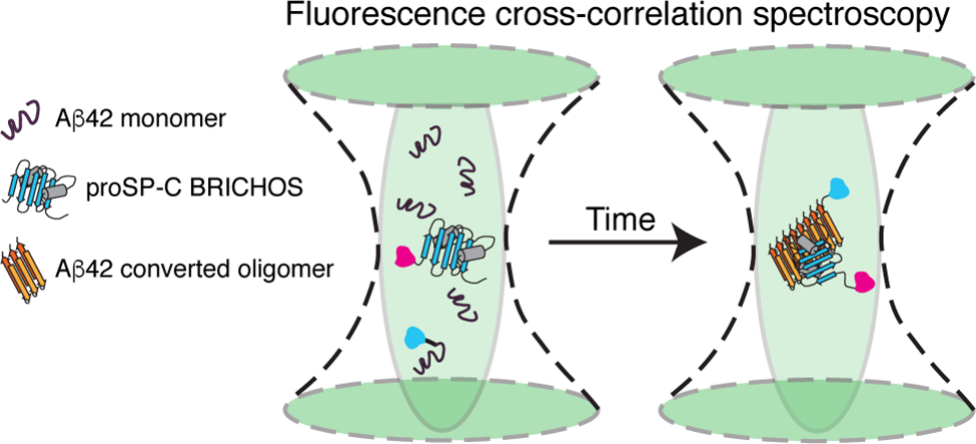

Protein oligomerization is a commonly encountered strategy by which the functional repertoire of proteins is increased. This, however, is a double-edged sword strategy because protein oligomerization is notoriously difficult to control. Living organisms have therefore developed a number of chaperones that prevent protein aggregation. The small ATP-independent molecular chaperone domain proSP-C BRICHOS, which is mainly trimeric, specifically inhibits fibril surface-catalyzed nucleation reactions that give rise to toxic oligomers during the aggregation of the Alzheimer’s disease-related amyloid-β peptide (Aβ42). Here, we have created a stable proSP-C BRICHOS monomer mutant and show that it does not bind to monomeric Aβ42 but has a high affinity for Aβ42 fibrils, using surface plasmon resonance. Kinetic analysis of Aβ42 aggregation profiles, measured by thioflavin T fluorescence, reveals that the proSP-C BRICHOS monomer mutant strongly inhibits secondary nucleation reactions and thereby reduces the level of catalytic formation of toxic Aβ42 oligomers. To study binding between the proSP-C BRICHOS monomer mutant and small soluble Aβ42 aggregates, we analyzed fluorescence cross-correlation spectroscopy measurements with the maximum entropy method for fluorescence correlation spectroscopy. We found that the proSP-C BRICHOS monomer mutant binds to the smallest emerging Aβ42 aggregates that are comprised of eight or fewer Aβ42 molecules, which are already secondary nucleation competent. Our approach can be used to provide molecular-level insights into the mechanisms of action of substances that interfere with protein aggregation.

Aggregation of protein into amyloid fibrils is associated with severe medical conditions, like Alzheimer’s disease, Parkinson’s disease, and type II diabetes.^1^ In particular, Alzheimer’s disease is linked to self-aggregation of the highly aggregation prone and toxic 42-residue variant of the amyloid-β peptide (Aβ42).^2^ While high-molecular weight Aβ fibrils appear to be relatively nontoxic, soluble low-molecular weight oligomers have emerged as the most toxic species.^3,4^ The recent development of chemical kinetic analysis revealed fundamental microscopic processes underlying the fibrillar aggregation of Aβ42 from supersaturated monomer solutions.^5^ In particular, the catalytic formation of new nuclei on the surface of amyloid fibrils, a process called secondary nucleation, appears to be the main culprit in the rapid formation of toxic oligomers.^6^ Despite great advances in understanding the kinetics of Aβ42 aggregation and structural investigations of Aβ42 oligomers,^7,8^ the sizes of aggregates that are able to catalyze secondary nucleation have not been experimentally determined. Here we employed the molecular chaperone domain proSP-C BRICHOS as a reporter for small, soluble, and secondary nucleation competent Aβ42 oligomers using fluorescence cross-correlation spectroscopy (FCCS). Because Aβ42 aggregation is a highly heterogeneous process, we applied the maximum entropy method for FCS (MEMFCS)^9^ fitting routine to analyze our data.

The proSP-C BRICHOS domain has shown a unique feature in exclusively blocking secondary nucleation reactions on the surface of Aβ42 fibrils.^10^ Therefore, it has served as a valuable model chaperone for the development of new approaches to study the kinetics of Aβ42 aggregation.^10−14^ However, in solution, the proSP-C BRICHOS domain exists as an equilibrium mixture between mainly trimers and monomers, where the former is thought to be an inactive storage conformation.^15,16^ To fluorescently label chaperone active proSP-C BRICHOS domain subunits, we rationally designed a stable monomeric variant by site-directed mutagenesis based on the trimeric proSP-C BRICHOS domain structure.^16^ The proSP-C BRICHOS monomer mutant, like the wild type (WT), does not bind Aβ42 monomers, has high affinity for Aβ42 fibrils, and specifically inhibits secondary nucleation. We were able to show that the proSP-C BRICHOS monomer mutant binds to a heterogeneous mixture of soluble Aβ42 species, and importantly, we measured binding to apparently secondary nucleation competent aggregates comprised of eight or fewer Aβ42 monomers.

## Results

### Design of a Stable ProSP-C BRICHOS Monomer Mutant

**Figure 1:**
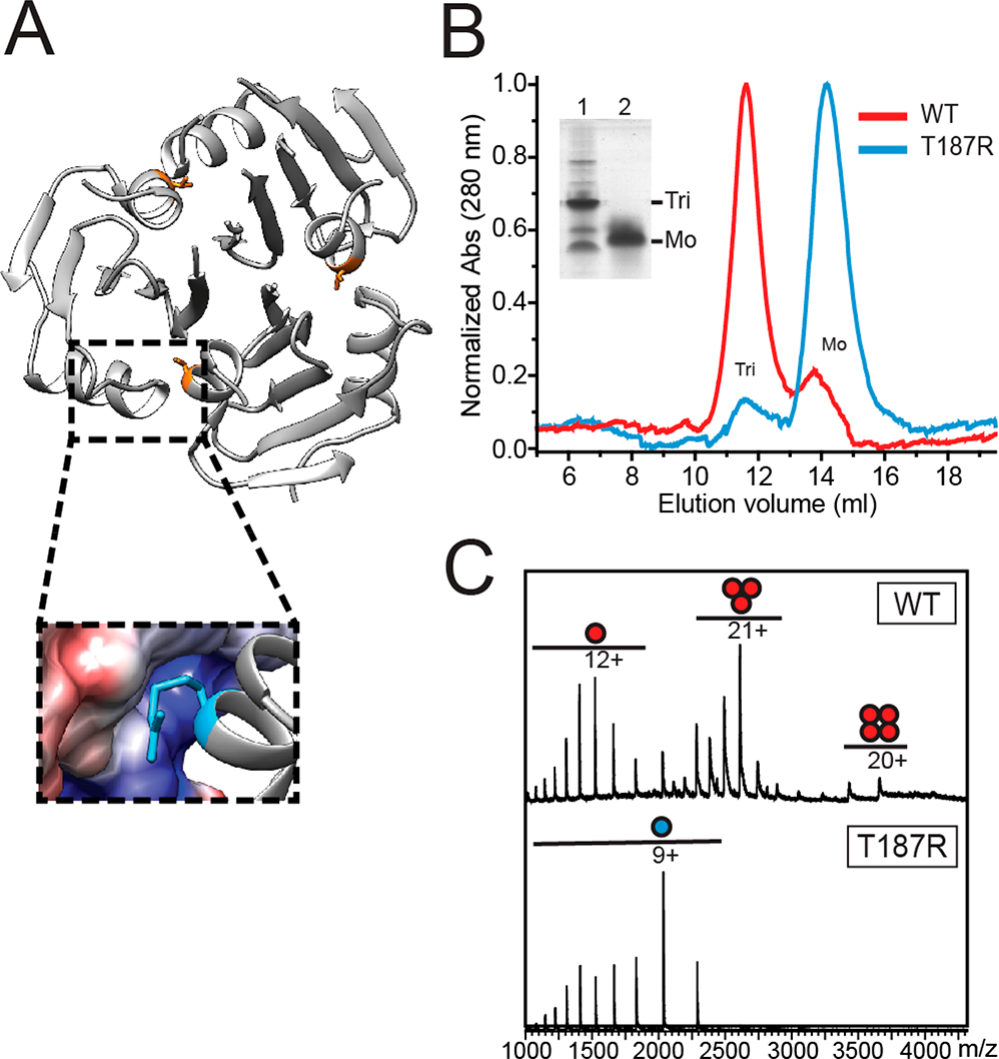
(A) Structure of the proSP-C BRICHOS WT homotrimer (Protein Data Bank entry 2YAD) with the side chain of Thr187 colored orange and close-up of one trimer subunit interface in which Thr at position 187 is mutated to Arg (blue). (B) SEC elution profiles of proSP-C BRICHOS WT (red) and proSP-C BRICHOS T187R (blue). The inset shows a native PAGE gel of samples prepared before separation by SEC for proSP-C BRICHOS WT (1) and proSP-C BRICHOS T187R (2), and the assembly states are indicated. (C) Native ESI-MS of proSP-C BRICHOS WT (red) and proSP-C BRICHOS T187R (blue) with quaternary structures and charge states indicated.

In the proSP-C BRICHOS homotrimer structure, we recognized a threonine pointing into a positively charged pocket of the neighboring domain in each subunit interface (Figure 1A). We substituted this threonine (T) with arginine (R) with the aim of causing steric hindrance and inducing charge repulsions between the trimer subunits (Figure 1A and Figure S1). Size exclusion chromatography (SEC) profiles and native polyacrylamide gel electrophoresis (PAGE) demonstrate that proSP-C BRICHOS T187R, in contrast to the WT, forms almost exclusively monomers (Figure 1B and Figure S1). Both proSP-C BRICHOS proteins have overall similar secondary structures, expose hydrophobic surfaces, and maintain their respective secondary and quaternary structures after overnight incubation at 37 °C as observed by circular dichroism (CD) spectroscopy and bis-ANS fluorescence (Figure S1). Electrospray ionization mass spectrometry (ESI-MS) confirms that proSP-C BRICHOS T187R is purely monomeric and the determined molecular mass of 18328 Da is in good agreement with the calculated mass of 18324 Da for the mutant monomer (Figure 1C).

### Effects of ProSP-C BRICHOS Variants on Aβ42 Fibrillation

To study the capacities of mainly trimeric proSP-C BRICHOS WT and the monomeric proSP-C BRICHOS T187R mutant to interfere with Aβ42 fibril formation, we performed bulk thioflavin T (ThT) fluorescence aggregation experiments. Under quiescent conditions, the aggregation of Aβ42 follows a sigmoidal growth curve that can be characterized by the reaction half-time (τ_1/2_) and maximum growth rate (*r*_max_). The data show that both proSP-C BRICHOS variants increase τ_1/2_ in a concentration-dependent manner, plateauing at a high relative proSP-C BRICHOS concentration, while *r*_max_ decreases exponentially (Figure S2). To compare equal numbers of proSP-C BRICHOS molecules, we assumed that proSP-C BRICHOS WT forms solely trimers in solution and corrected the concentration accordingly (Figure S2). While the delay of τ_1/2_ and the decrease in *r*_max_ at low BRICHOS:Aβ42 ratios can be explained by the molecular stoichiometry, the effects on both fitting parameters are somewhat more pronounced for monomeric proSP-C BRICHOS T187R at high BRICHOS concentrations.

### Binding Affinities for Monomeric and Fibrillar Aβ42

**Figure 2:**
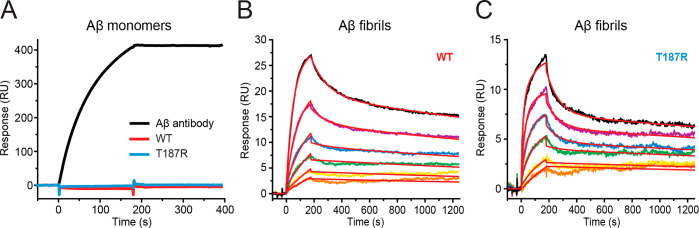
SPR sensorgrams of proSP-C BRICHOS binding to Aβ42 monomers or fibrils. (A) 0.1 μM Aβ antibody (6E10; black), 50 μM proSP-C BRICHOS WT (red), and 50 μM proSP-C BRICHOS T187R (blue) binding to immobilized Aβ42 monomers. (B) ProSP-C BRICHOS WT and (C) proSP-C BRICHOS T187R binding to immobilized Aβ42 fibrils fitted to a heterogeneous ligand model (BRICHOS concentrations of 0.391, 0.781, 1.56, 3.13, 6.25, and 12.5 μM in orange, yellow, green, blue, violet, and black, respectively). Global fits are colored red. Data sets were collected twice with similar results.

Next, we set out to investigate proSP-C BRICHOS WT and proSP-C BRICHOS T187R affinities for monomeric or fibrillar Aβ42 using surface plasmon resonance (SPR) (Figure 2). The presence of immobilized Aβ42 species was confirmed by using a monoclonal antibody directed against Aβ, while carbonic anhydrase served as a negative control for unspecific binding to fibrils (Figure 2 and Figure S3). We were not able to detect any specific binding to monomeric Aβ42 of either WT, which is in line with previous findings,^10^ or proSP-C BRICHOS T187R using concentrations of ≤50 μM (Figure 2A). The absence of binding between monomeric Aβ42 and proSP-C BRICHOS T187R was confirmed by FCS and FCCS (Figure S4). FCS revealed no changes in the diffusion of Aβ42 monomers fluorescently labeled with HiLyteFluor488 (HiLyteFluor488-Aβ42) in the presence of different amounts of unlabeled proSP-C BRICHOS T187R (10:1, 1:1, and 1:10), but the diffusion time remained the same as for HiLyteFluor488-Aβ42 alone, τ_D,Aβ 42_ (70 ± 10) μs. Dual-color FCCS using HiLyteFluor488-Aβ42 and proSP-C BRICHOS T187R fluorescently labeled with Atto655 (proSP-C BRICHOS T187R-Atto655) further corroborated that there are no detectable interactions between monomeric Aβ42 and proSP-C BRICHOS T187R (Figure S4). This is evident from the unchanged, within experimental error, characteristic decay times of the temporal autocorrelation curves (ACCs) for monomeric HiLyteFluor488-Aβ42 (τ_D,Aβ 42_ = (70 ± 10) μs) and monomeric proSP-C BRICHOS T187R-Atto655 (τ_D,T187R_ = (175 ± 35) μs) in mixed preparations (Figure S4), as well as the lack of cross-correlation between the two signals even at a 1:10 Aβ42:proSP-C BRICHOS T187R ratio (Figure S4). A detailed analysis of cross-talk effects is provided in the Methods in the Supporting Information.

In contrast to the lack of interactions with Aβ42 monomers, both proSP-C BRICHOS proteins have a high apparent binding affinity for Aβ42 fibrils, determined by SPR. The kinetics of binding to immobilized fibrils fit well to a global heterogeneous ligand binding model with two binding sites (Figure 2B,C). The model was confirmed using linear regression analysis of the association phase separately for low and high concentrations (data not shown). We thus determined, for both BRICHOS variants, two apparent *K*_D_ values, one in the micromolar range (*K*_D1_) and one in the nanomolar range (*K*_D2_) (Table 1). The apparent strong binding affinity (*K*_D2_) of proSP-C BRICHOS T187R for Aβ42 fibrils is approximately 5-fold greater than that of proSP-C BRICHOS WT. The dissociation rate constant of the second component (*k*_d2_) measured for proSP-C BRICHOS WT is in good agreement with previously published values in which only one proSP-C BRICHOS concentration was used to determine the kinetic parameters (Table 1).^10^ However, we measured a somewhat slower association rate for proSP-C BRICHOS WT, explaining the discrepancy between the apparent *K*_D2_ of ≈130 nM and the published *K*_D_ of ≈40 nM.^10^ The second binding site is likely due to unspecific binding or represents an ∼1000-fold lower affinity binding site on Aβ42 fibrils. Importantly, the maximum measured response units under equal conditions are higher for proSP-C BRICHOS WT than for proSP-C BRICHOS T187R at all measured concentrations (Figure 2B,C) and at high analyte concentrations differ by a factor of ∼3 (data not shown). This indicates that monomeric proSP-C BRICHOS T187R and proSP-C BRICHOS WT trimers bind to Aβ42 fibrils.

**Table 1:** Kinetic Rate Constants and Apparent Binding Constants Determined by SPR for the Interaction of Fibrillar Aβ42 with ProSP-C BRICHOS WT and ProSP-C BRICHOS T187R Using a Heterogeneous Ligand Binding Model (corresponding data shown in Figure 2)^a^

	*k*_a1_ (M^–1^ s^–1^)	*k*_d1_ (s^–1^)	*K*_D1_ (μM)	*k*_a2_ (M^–1^ s^–1^)	*k*_d2_ (s^–1^)	*K*_D2_ (nM)
WT	175 ± 35	(85.3 ± 0.4) × 10^–4^	49 ± 12	1770 ± 24	(2.30 ± 0.02) × 10^–4^	130 ± 3
T187R	88 ± 4	(112.0 ± 1.0) × 10^–4^	128 ± 8	6800 ± 110	(1.70 ± 0.03) × 10^–4^	25 ± 1
WT*	–	–	–	5100	2.1 × 10^–4^	≈40

a Errors correspond to the fitting error. Values for WT* determined by Cohen et al.^10^

### ProSP-C BRICHOS T187R Suppresses Secondary Nucleation

Inhibition of Aβ42 fibril formation by different classes of molecular chaperones is known to be associated with their interference with different microscopic processes during self-aggregation.^17^ The WT proSP-C BRICHOS domain is a highly specific secondary nucleation inhibitor under quiescent conditions.^10,17^ Consequently, we investigated if the effect on the Aβ42 aggregation mechanism is similar for the monomeric proSP-C BRICHOS T187R mutant, using a bulk ThT fluorescence assay. We performed a series of kinetic experiments and fitted our data with the analytical solution that describes the time evolution of Aβ42 fibril formation as a function of several microscopic rate constants, i.e., primary nucleation (*k*_n_), secondary nucleation (*k*_2_), and elongation (*k*_+_).^5^

**Figure 3:**
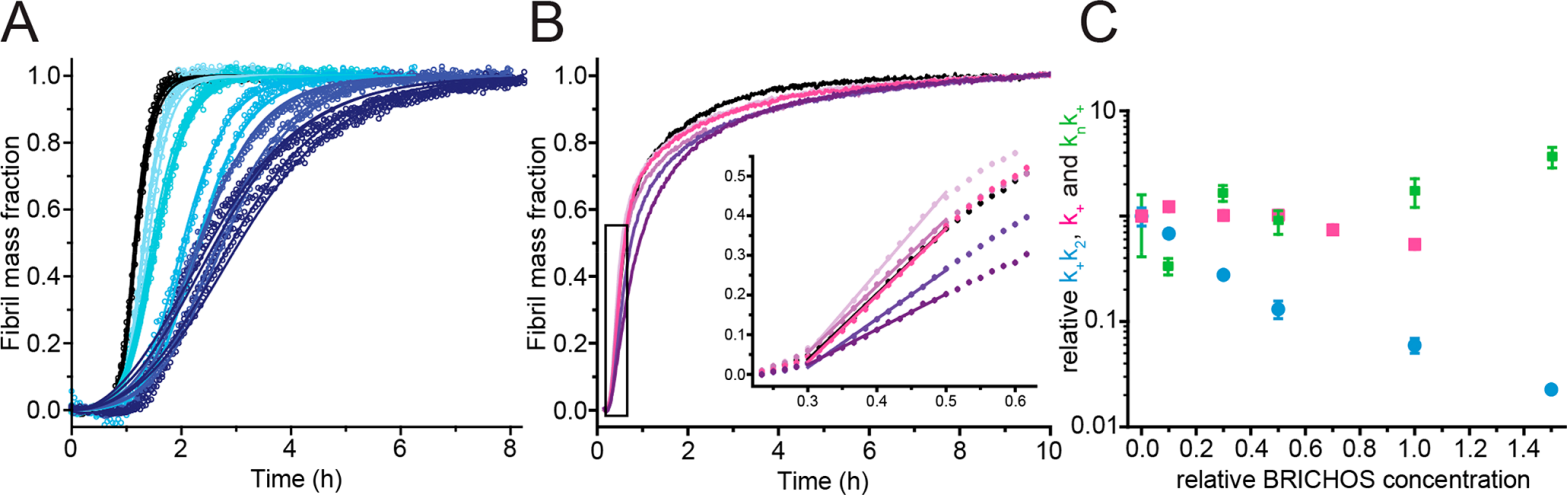
Aβ42 fibrillation kinetics in the presence of proSP-C BRICHOS T187R. (A) Individual fits (colored lines) of normalized aggregation traces of 3 μM Aβ42 alone (black) and in the presence of 0.1, 0.3, 0.5, 1, and 1.5 molar equivalents of proSP-C BRICHOS T187R (light blue to dark blue empty circles) using a kinetic nucleation model in which the combined rate constants  and  are free fitting parameters. (B) Seeded reaction profiles of 3 μM Aβ42 with 0.6 μM preformed fibrils (black) in the presence of 0.1, 0.3, 0.5, 0.7, and 1.0 molar equivalent of proSP-C BRICHOS T187R (light pink to dark purple). Data represent the average of four replicates. The black rectangle shows a close-up of the onset of aggregation. Colored lines are corresponding fits to the data using a linear regression. (C) Effects of relative rate constants *k*_n_*k*_+_ (green), *k*_+_ (pink), and *k*_+_*k*_2_ (blue) from the fits shown in panels A and B. Rates have been normalized to Aβ42 in the absence of BRICHOS. Data represent the mean ± the standard deviation of four or five replicates.

Aβ42 fibril formation under quiescent conditions has been described to be dominated by monomer-dependent surface-catalyzed secondary pathways, indicated by a characteristic γ-exponent of approximately −1.3.^5^ To test if the aggregation of Aβ42 in the presence of proSP-C BRICHOS T187R is dominated by secondary nucleation, we recorded the aggregation of varying Aβ42 monomer concentrations in the absence and presence of a constant proSP-C BRICHOS T187R concentration. We found that the γ exponent is similar with and without proSP-C BRICHOS T187R (−1.32 ± 0.05 and −1.41 ± 0.06, respectively), suggesting that the underlying Aβ42 aggregation mechanism is in both cases monomer-dependent secondary nucleation (Figure S5). Next, we investigated the effects of proSP-C BRICHOS T187R on the microscopic rate constants of Aβ42 aggregation. The reaction profiles obtained at a constant Aβ42 concentration and varying BRICHOS concentrations were fitted with the combined rate constants for primary  and secondary  nucleation pathways being free fitting parameters (Figure 3A). To decouple nucleation rates from fibril end elongation,^18^ we measured Aβ42 fibrillation kinetics with 20% preformed seeds in the absence and presence of increasing proSP-C BRICHOS T187R concentrations (Figure 3B). The results show that proSP-C BRICHOS T187R affects primarily secondary nucleation pathways and to some minor extent fibril end elongation, which is obvious only at equimolar concentrations (Figure 3C).

Global fit analysis of the aggregation traces obtained with varying Aβ42 monomer concentrations in the absence and presence of 0.3 molar equivalent of proSP-C BRICHOS T187R, where the fitting parameters  and  are constrained to the same value across all Aβ42 peptide concentrations, suggests that effects on the aggregation pathways are correlated to primary and secondary nucleation processes (Figure S5 and Table S1). To study which microscopic rate constant is predominantly affected by proSP-C BRICHOS T187R, i.e., *k*_n_, *k*_2_, or *k*_+_, we fitted the data sets with constant Aβ42 concentrations and varying proSP-C BRICHOS T187R concentrations globally, where only a single parameter is allowed to vary freely across all BRICHOS concentrations, while the two others are fixed to the value of Aβ42 alone. Our results indicate that the global fits describe the data sets best with *k*_2_ as the sole free fitting parameter [residual sum of squares (RSS) =0.8] compared to *k*_n_ (RSS = 4.3) or *k*_+_ (RSS = 1.5) (Figure S6). Suppression of secondary nucleation mechanisms is associated with a decrease in the level of formation of Aβ42 oligomer intermediates.^10^ Therefore, we simulated the time evolution of the nucleation rate based on the parameters determined from (i) global fits of different Aβ42 concentrations with and without 0.3 molar equivalent of BRICHOS (Figure S5) and (ii) individual fits of 3 μM Aβ42 with and without varying molar equivalents of BRICHOS (Figure 3A). For our calculations, we used elongation rates from seeded fibrillation experiments obtained at matching BRICHOS concentrations (Figure 3B). We find that 0.3 molar equivalent of proSP-C BRICHOS T187R decreases the relative number of generated Aβ42 nucleation units by ∼60% (Figure S7). Importantly, this reduction is consistent with both individual calculations. Taken together, our data show that monomeric proSP-C BRICHOS T187R inhibits mainly secondary nucleation events, similar to the WT, and thereby reduces the number of surface-catalyzed nuclei.

### ProSP-C BRICHOS T187R Binds Soluble Aβ42 Aggregates of Different Sizes

Having established how monomeric proSP-C BRICHOS T187R inhibits Aβ42 fibril formation, we set out to investigate interactions with soluble Aβ42 aggregates. To this aim, we used FCCS and monitored the time course of Aβ42 aggregation in solutions containing different initial concentrations of unlabeled Aβ42 (5, 10, or 20 μM) in the presence of 100 nM HiLyteFluor488-Aβ42, and 483 nM proSP-C BRICHOS T187R, of which 100 nM was fluorescently labeled with Atto655 (proSP-C BRICHOS T187R-Atto655). In this way, the Aβ42:proSP-C BRICHOS T187R ratio was 10.5:1, 21:1, and 41.5:1 for experiments with 5, 10, and 20 μM unlabeled Aβ42, respectively, allowing us to examine in detail the interactions of proSP-C BRICHOS T187R with Aβ42 aggregates of different sizes formed throughout the aggregation process.

**Figure 4:**
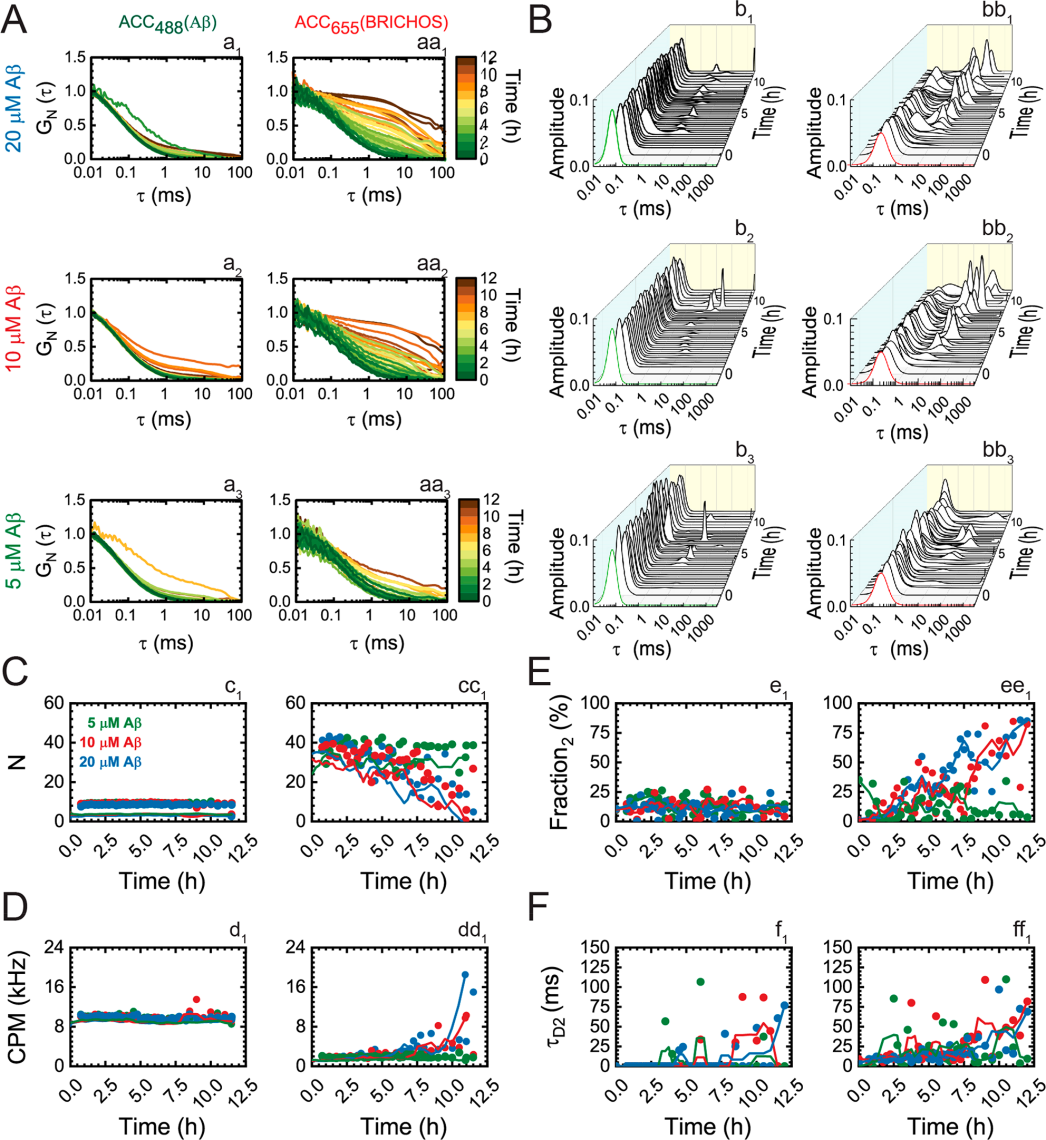
ProSP-C BRICHOS T187R binding to soluble Aβ42 aggregates of different sizes; analysis of ACCs in FCCS measurements. (A) Normalized temporal ACCs of 100 nM HiLyteFluor488-Aβ42 (a_1_–a_3_) and 100 nM proSP-C BRICHOS T187R-Atto655 (aa_1_–aa_3_) during Aβ42 aggregation for different initial concentrations of unlabeled Aβ42 [(1) 20 μM, (2) 10 μM, and (3) 5 μM] in the presence of unlabeled 385 nM proSP-C BRICHOS T187R. (B) MEMFCS analysis of ACCs shown in panel A displaying the distribution of diffusion times for 100 nM HiLyteFluor488-Aβ42 (b_1_–b_3_) and 100 nM proSP-C BRICHOS T187R-Atto655 (bb_1_–bb_3_) during Aβ42 aggregation. The green and red diffusion time distributions reflect the diffusion of monomeric HiLyteFluor488-Aβ42 and proSP-C BRICHOS T187R-Atto655, respectively. (C) Apparent average number of HiLyteFluor488-Aβ42 (c_1_) and proSP-C BRICHOS T187R-Atto655 (cc_1_) molecules in the observation volume element during the time course of Aβ42 aggregation. (D) Apparent brightness of HiLyteFluor488-Aβ42 (d_1_) and proSP-C BRICHOS T187R-Atto655 (dd_1_) molecules, as reflected by the counts per molecule and second (CPM). (E) Relative amplitude of the second component for HiLyteFluor488-Aβ42 (e_1_) and proSP-C BRICHOS T187R-Atto655 (ee_1_) molecules. (F) Diffusion time of the second component of HiLyteFluor488-Aβ42 (f_1_) and proSP-C BRICHOS T187R-Atto655 (ff_1_) molecules during the time course of Aβ42 aggregation.

ACCs for HiLyteFluor488-Aβ42 and proSP-C BRICHOS T187R-Atto655 showed that proSP-C BRICHOS T187R-Atto655 binds to soluble Aβ42 aggregates of different sizes (Figure 4A). This is evident from the change in the shape of the ACCs, which gradually lose their sigmoid shape that is characteristic of monodisperse systems, and the appearance of one or more components with characteristic decay times longer than the characteristic decay time measured at the beginning of the reaction. MEMFCS analysis of the ACCs (Figure 4B) showed that during the course of Aβ42 aggregation the single-component distribution of diffusion times for HiLyteFluor488-Aβ42 and proSP-C BRICHOS T187R-Atto655, which is characteristic for time zero (Figure 4B, green and red), becomes bimodal at later time points for the majority of measurements (Figure 4B, black). For both species, the diffusion time of the first component (τ_D1_) was within experimental error indistinguishable from the diffusion time of the free monomeric species (τ_D1,488_ = τ_D,Aβ42_ = (70 ± 10) μs for HiLyteFluor488-Aβ42, and τ_D1,633_ = τ_D,T187R_ = (175 ± 35) μs for proSP-C BRICHOS T187R-Atto655). The diffusion time of the first component did not change during the course of Aβ42 aggregation, indicating that a fraction of unbound HiLyteFluor488-Aβ42 and proSP-C BRICHOS T187R-Atto655 was always present in the reaction mixture, whereas the diffusion time of the second component increased over time (Figure 4B). Of note, several measurements in which aggregates with diffusion times of >200 ms were observed were not included in the analysis. For these very large aggregates, it takes on average ∼0.2 s to pass through the observation volume element. This diffusion time is long compared to the signal acquisition time (10 s). Hence, the ACCs do not decay to 1. Such ACCs could be occasionally observed but were excluded from the analysis. In line with these results obtained by the MEMFCS analysis, all ACCs were refitted using a three-dimensional (3D) diffusion model for two components (*n* = 2) in eq 8 (Methods), where the diffusion time of the first component was fixed to the value of the respective monomeric species, whereas the diffusion time (τ_D2_) and the relative amplitude of the second component (*f*_2_ = 1 – *f*_1_) were allowed to freely change. The results of the two-component analysis of ACCs, summarized in Figure 4C–F, reveal important differences in the involvement of HiLyteFluor488-Aβ42 and proSP-C BRICHOS T187R-Atto655 in the Aβ42 aggregation process.

We first note the striking difference between the simultaneously acquired ACC_488_ [Figure 4A (a_1_–a_3_)] and ACC_633_ [Figure 4A (aa_1_–aa_3_)]. Most notably, the relative contribution of the second component in the ACC_488_ is low, while it is steadily increasing in ACC_633_. This is because HiLyteFluor488-Aβ42 is readily displaced by unlabeled monomeric Aβ42 that is present in large excess in the reaction mixture (1:50, 1:100, and 1:200 for 5, 10, and 20 μM Aβ42, respectively). As a consequence, a large proportion of HiLyteFluor488-Aβ42 remained unbound and the average number of HiLyteFluor488-Aβ42 molecules in the observation volume element (OVE) [Figure 4C (c_1_)] and the brightness of HiLyteFluor488-Aβ42 molecules, as reflected by the counts per molecule and second {CPM_488_ [Figure 4D (d_1_)]}, remained unchanged over time. In contrast, proSP-C BRICHOS T187R-Atto655 for which the ratio of labeled versus unlabeled proSP-C BRICHOS T187R molecules was 1:3, could readily bind to the Aβ42 aggregates, and the likelihood that more than one proSP-C BRICHOS T187R-Atto655 molecule could bind to the same Aβ42 aggregate was not significantly decreased by the unlabeled fraction. Consequently, as one can clearly see from the apparent decrease in the number of proSP-C BRICHOS T187R-Atto655 molecules [Figure 4C (cc_1_)] and the strong increase in brightness at advanced stages of the Aβ42 aggregation process [Figure 4D (dd_1_)], more than one proSP-C BRICHOS T187R-Atto655 molecule could bind to the same Aβ42 aggregate. This is particularly visible for high initial Aβ42 concentrations (10 and 20 μM), where up to 10 proSP-C BRICHOS T187R-Atto655 molecules bound to the same large Aβ42 aggregate, which can be clearly seen from the brightness that increased from CPM = 2 kHz for small/medium-sized Aβ42 aggregates characterized by τ_D_ < 5 ms to CPM = 20 kHz for Aβ42 aggregates characterized by τ_D_ = 75 ms. Furthermore, due to the intensive displacement of HiLyteFluor488-Aβ42 by unlabeled Aβ42, the fraction of bound HiLyteFluor488-Aβ42 molecules, i.e., the relative amplitude of the second component (*f*_2_), was always lower for HiLyteFluor488-Aβ42 than for proSP-C BRICHOS T187R-Atto655 molecules [Figure 4A (a_i_ vs aa_i_) and Figure 4E (e_1_ vs ee_1_)]. Finally, the size of Aβ42 aggregates at the end of observation (t_end_ = 12.0 h) was somewhat smaller when the initial Aβ42 concentration was the lowest (5 μM), i.e., when the Aβ42:proSP-C BRICHOS T187R ratio was the lowest and the capacity of proSP-C BRICHOS T187R to inhibit Aβ42 aggregation was the largest, which can be clearly seen from the diffusion times that for most of the time remained <50 ms (Figure 4F).

**Figure 5:**
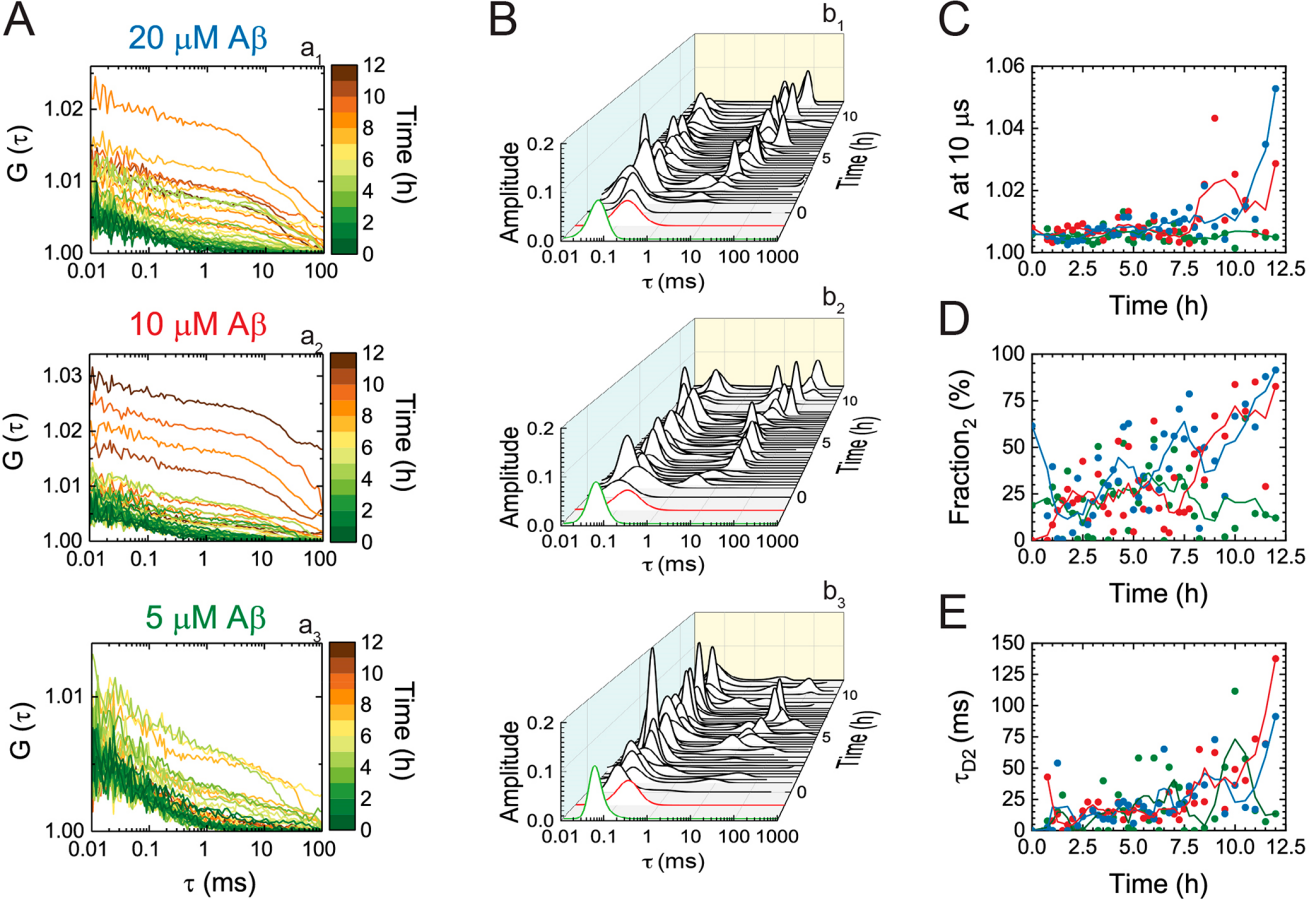
ProSP-C BRICHOS T187R binding to soluble Aβ42 aggregates of different sizes; analysis of CCCs in FCCS measurements. (A) CCCs of Aβ42 aggregates in complex with proSP-C BRICHOS T187R-Atto655. (B) MEMFCS analysis of CCCs shown in panel A reflecting changes in the distribution of diffusion times. The green and red diffusion time distributions represent the diffusion of HiLyteFluor488-Aβ42 (ACC_488_) and proSP-C BRICHOS T187R-Atto655 (ACC_633_), respectively, at the beginning of the reaction (*t* = 0 h). (C) Amplitude of the CCCs during Aβ42 aggregation. (D) Relative amplitude of the second component that increases over time. (E) Size of dually labeled aggregates, as reflected by changes in the diffusion time of the second component.

The concomitantly acquired CCCs (Figure 5A) and their analysis by MEMFCS (Figure 5B) also showed two principal decay times. Fitting the CCCs by the equation for free 3D diffusion of two components (eq 8) without the triplet state term [*n* = 2 (Methods)] revealed that the cross-correlation amplitude (Figure 5C), the relative amplitude of the second component (Figure 5D), and the diffusion time of the second component (Figure 5E) all gradually increase during the course of Aβ42 aggregation, reflecting the accumulation of proSP-C BRICHOS T187R-Atto655 complexes with HiLyteFluor488-Aβ42-labeled Aβ42 oligomers of different sizes.

### ProSP-C BRICHOS T187R Binds Small Soluble Aβ42 Aggregates

The largely bimodal distribution of diffusion times observed for HiLyteFluor488-Aβ42 and proSP-C BRICHOS T187R-Atto655 molecules (Figure 4) is easy to understand intuitively, as it reflects the existence of a dynamic equilibrium between free molecules and molecules bound to Aβ42 aggregates of various, ever-increasing sizes. However, the presence of two principally distinct diffusion times in the CCCs (Figure 5B) is puzzling and cannot be explained using the same reasoning. This motivated us to examine the CCCs in more detail, to establish whether the characteristic decay time at short lag times (τ_D1_) is solely due to signal bleed-through from the green to the red channel (Figure S4) or if this component reflects true interactions between proSP-C BRICHOS T187R-Atto655 and HiLyteFluor488-Aβ42-labeled Aβ42 oligomers. As a first step, we used paired *t* test analysis to compare the short decay time of the CCCs, τ_D1_,_CCC_ in a time series, with the short decay times of the simultaneously recorded ACCs, τ_D1,488_ (HiLyteFluor488-Aβ42) and τ_D1,633_ (proSP-C BRICHOS T187R-Atto655). We observed that τ_D1,488_ < τ_D1,CCC_ < τ_D1,633_, with τ_D1,CCC_ being significantly longer than τ_D1,488_ (*t* > 2.91575; *p* < 0.005). We also observed that the effect size was dependent on the concentration of Aβ42 and was largest for 5 μM Aβ42 (*t* and *p* values for all Aβ42 concentrations are given in Methods). This prompted us to further examine the CCCs acquired in measurements with 5 μM Aβ42.

**Figure 6:**
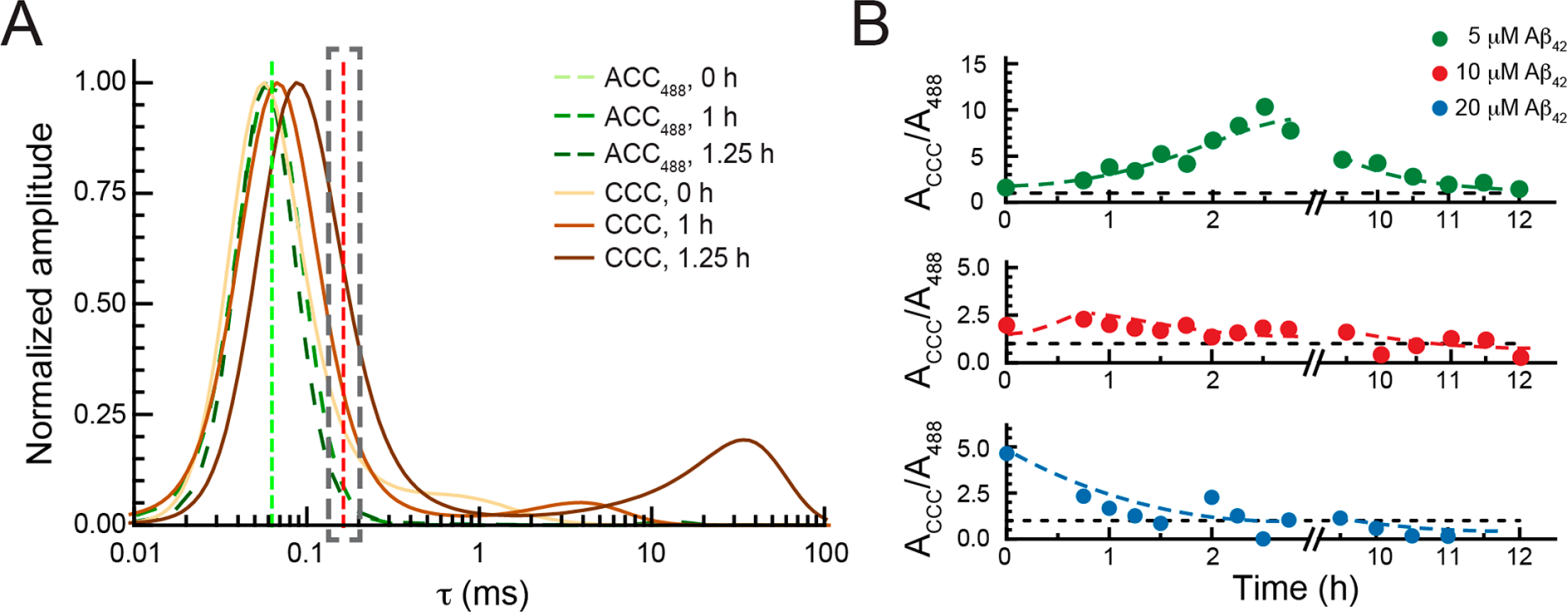
ProSP-C BRICHOS T187R binding to small soluble Aβ42 aggregates; analysis of CCCs in FCCS measurements. (A) Normalized MEMFCS diffusion time distributions shown in Figure 4B (b_3_) and Figure 5B (b_3_) for HiLyteFluor488-Aβ42 (ACC_488_, green) and the corresponding CCCs, reflecting the presence of dually labeled Aβ42 aggregates in a complex with proSP-C BRICHOS T187R-Atto655 (brown) at 0 h (lightest), 1 h (darker), and 1.25 h (darkest). Green and red vertical dashed lines indicate the diffusion times of HiLyteFluor488-Aβ42 and proSP-C BRICHOS T187R-Atto655, respectively. (B) Time evolution of the amplitude of the CCC relative to the amplitude of the ACC_488_ (*A*_CCC_/*A*_488_) at τ_D1,633_ (indicated by the dashed gray rectangle in panel A) for 5 μM (green), 10 μM (red), and 20 μM (blue) Aβ42. Adjacent averaging of two data points is applied to smooth out short-term fluctuations and highlight long-term trends. The dashed lines are drawn to guide the eye.

Comparison of the MEMFCS-derived distributions of diffusion times at different time points during the aggregation of 5 μM Aβ42 (Figure 6A and Figure S8) revealed that the distribution profiles of the ACCs488 are Gaussian. This is expected because a large portion of HiLyteFluor488-Aβ42 is unbound and freely diffusing as it is displaced by unlabeled Aβ42, which is present in large excess. In contrast, the distributions of diffusion times for the corresponding CCCs show a positive skew toward longer lag times. Consequently, for lag times that are longer than τ_D1,488_, the amplitude of the CCC is larger than the amplitude of the corresponding ACC488 (Figure 6A and Figure S8). These results show that the short characteristic decay times of the CCCs contain contributions from both, the signal bleed-through from the green to the red channel, which defines to a very large extent the position of the peak but also indicates true interactions between proSP-C BRICHOS T187R-Atto655 and HiLyteFluor488-Aβ42-labeled Aβ42 oligomers, which can be clearly seen from the positive skew of the CCCs that builds up as the cross-correlation signal increases above the background from the signal bleed-through of green-labeled molecules.

Furthermore, we observed a positive cross-correlation signal, which is well above the signal bleed-through background, at τ_D1,633_, the diffusion time of monomeric proSP-C BRICHOS T187R-Atto655 (Figure 6A, red dashed line, and Figure S8). This suggests that complexes of proSP-C BRICHOS T187R-Atto655 with Aβ42 aggregates form and are sufficiently small to not significantly change the distribution of the diffusion time of proSP-C BRICHOS T187R-Atto655 monomers. The molecular weight of monomeric Aβ42 is 4.5 kDa, and proSP-C BRICHOS T187R-Atto655 monomers have a molecular weight of 18.8 kDa. Given that the diffusion time of spherical molecules scales with the third power of the molecular weight, an Aβ42 oligomer that is bound by one proSP-C BRICHOS T187R-Atto655 monomer and gives rise to a cross-correlation signal but does not change the diffusion time of the aggregate to a measurable extent must be smaller than a proSP-C BRICHOS T187R-Atto655 trimer (56.4 kDa). Binding of one proSP-C BRICHOS T187R-Atto655 monomer to an Aβ42 oligomer that contains eight or fewer Aβ42 monomers would give τ_D1,633_ ≈ τ_D1,CCC_, and hence, they are indistinguishable within the error of the measurement.

To test this interaction, we have examined how the relative cross-correlation amplitude, i.e., the amplitude of the CCC relative to the amplitude of the ACC488 at τ_D1,633_, changes over time depending on the total concentration of Aβ42 (Figure 6B). Here the relative cross-correlation amplitude at τ_D1,633_, which equals the number of doubly labeled complexes between proSP-C BRICHOS T187R-Atto655 and small HiLyteFluor488-Aβ42-labeled Aβ42 oligomers that consist of two to eight monomers (*N*_RG_), is related to the total number of molecules carrying a red label (*N*_R,tot_ = *N*_RG_ + *N*_R_).^19^ For 5 μM Aβ42, where the aggregation compared to the other concentrations is the slowest and the ratio of labeled to unlabeled Aβ42 is the highest, a transient increase in the number of complexes between one proSP-C BRICHOS T187R-Atto655 and small Aβ42 oligomers is observed. This complex formation reaches a maximum value after approximately 3–3.5 h and slowly decreases thereafter (Figure 6B, green). At higher Aβ42 concentrations, the onset at which the maximum number of complexes between proSP-C BRICHOS T187R-Atto655 and small HiLyteFluor488-Aβ42-labeled Aβ42 oligomers that consist of two to eight monomers occurs shifts to earlier time points (Figure 6B). At the highest Aβ42 concentration, these complexes form right at the start of the reaction and decrease over time as the equilibrium shifts toward large complexes consisting of several proSP-C BRICHOS T187R-Atto655 molecules and large HiLyteFluor488-Aβ42-labeled Aβ42 oligomers (Figure 6B, blue).

## Discussion

In the past few years, the detailed kinetic mechanisms and dynamic turnover of aggregation intermediates during Aβ42 fibril formation have been investigated using a variety of biochemical and spectroscopic techniques.^20−24^ Molecular chaperones, e.g., clusterin and αB-crystallin, have been shown to interact with and stabilize Aβ oligomers, ranging from dimers to 50-mers.^22,25^ However, clusterin shows effects on primary and secondary nucleation, and αB-crystallin associates with fibril ends,^26,27^ which is in contrast to the proSP-C BRICHOS domain, which exclusively inhibits secondary nucleation.^10^ This motivated us to create and carefully characterize a FCS-compatible monomeric variant of the molecular chaperone domain proSP-C BRICHOS using multiple complementary biochemical and biophysical methods and determine binding to small soluble Aβ42 oligomers by FCS. Because the analysis of heterogeneous aggregation processes is inherently difficult, we used the MEMFCS, a fitting procedure developed to resolve FCS data based on a quasicontinuous distribution of highly heterogeneous diffusing components.^9^ Our studies reveal that Aβ42 oligomers comprised of eight or fewer monomers have already developed a secondary nucleation competent structure.

**Figure 7:**
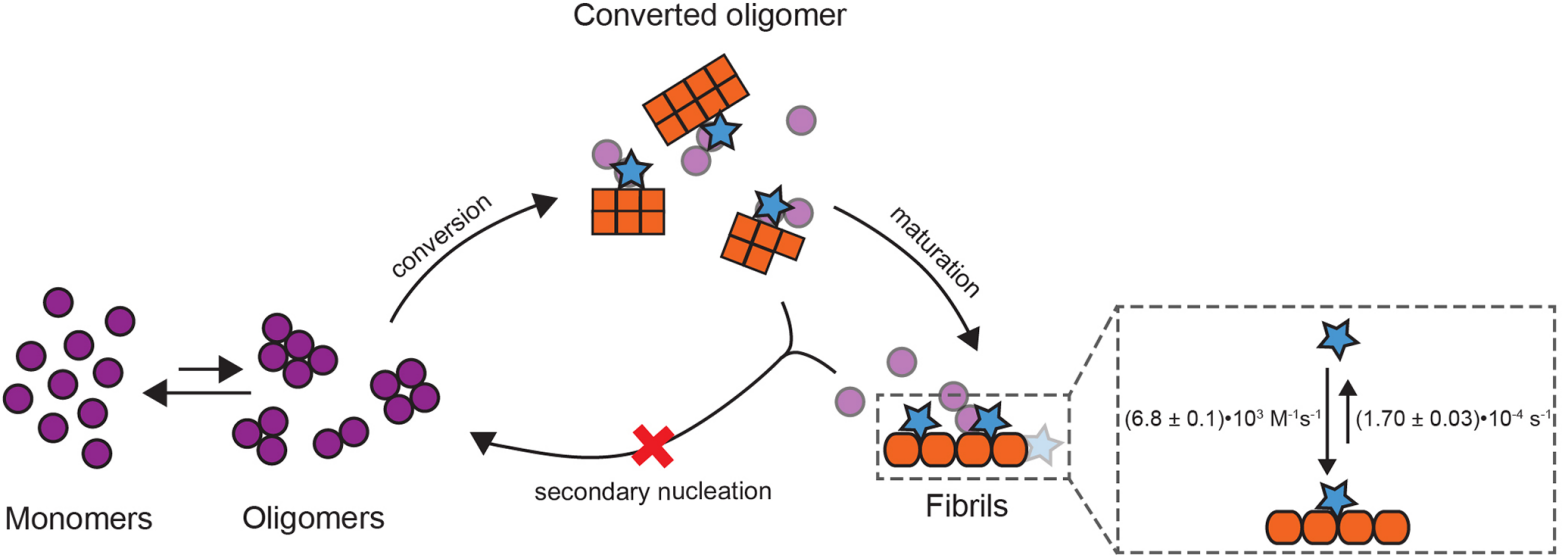
Model of proSP-C BRICHOS T187R interactions during the catalytic cycle of Aβ42 self-aggregation. Monomeric proSP-C BRICHOS (blue stars) does not bind to monomeric or early oligomeric Aβ42 (purple circles) but to converted oligomers that consist of eight or fewer Aβ42 molecules and have acquired secondary nucleation competent structures (orange squares). Throughout this reaction, monomeric proSP-C BRICHOS T187R inhibits secondary nucleation pathways (red cross), thereby reducing the number of newly formed toxic oligomers.

Recent experimental data combined with mathematical modeling to simulate the dynamics of oligomer formation and conversion showed that Aβ42 monomers assemble into a heterogeneous mixture of small, more unstable oligomers and converting oligomers that are more likely to transform into fibrillar structures.^11^ Remarkably, the simulated size ranges for these oligomer species (between 2- and 9-mers)^11^ overlap very well with the smallest Aβ42 aggregates with which the proSP-C BRICHOS monomer mutant interacts. The resolution limit in our experimental setup does not allow us to distinguish between smaller, supposedly more unstable oligomers (2–4-mers) and somewhat larger converting or just converted oligomers (5–9-mers).^11^ Nevertheless, direct interactions of the BRICHOS domain with early unstable oligomers would result in slower rate constants for primary nucleation, while interactions with fibrillar structures that just converted from oligomers would result in effects on secondary nucleation and/or elongation. We found that the proSP-C BRICHOS monomer mutant does not interact with Aβ42 monomers, has no effects on primary nucleation, but strongly associates with Aβ42 fibrils and efficiently prevents secondary nucleation pathways during Aβ42 fibrillation. From this, we conclude that the proSP-C BRICHOS monomer mutant binds to Aβ42 aggregates that consist of eight or fewer Aβ42 monomers that are already secondary nucleation potent (Figure 7). Furthermore, the high efficiency of the monomeric proSP-C BRICHOS mutant to prevent surface-catalyzed secondary nucleation is likely related to the ability to bind the smallest emerging fibrillar structures. Therefore, our data imply that the simulated sizes for converting oligomers^11^ are at the verge of fibril-forming structures.

The proSP-C BRICHOS structure is composed of a central five-stranded β-sheet that is flanked by two α-helices.^16^ Many hydrophobic residues are located on face A of this central β-sheet, and it was suggested that it is complementary to the substrates.^28^ Recently, a high-resolution structure of an Aβ42 tetramer in a lipid environment was reported.^7^ This Aβ42 tetramer forms a six-stranded β-sheet core that almost exclusively contains hydrophobic residues.^7^ We speculate that the BRICHOS domain can expose face A and bind to the hydrophobic core region of, e.g., soluble Aβ42 tetramers. This would also imply that already the six-stranded β-sheet core of an Aβ42 tetramer can promote secondary nucleation reactions. Furthermore, high-resolution structures of mature Aβ42 fibrils show a cluster of hydrophobic residues that are buried in the intermolecular contact site between two Aβ42 protofibrils.^29−31^ We hypothesize that this hydrophobic cluster could be surface-exposed in small secondary nucleation competent aggregates and hence constitutes a potential binding site for proSP-C BRICHOS on small pre- and protofibrillar aggregates.

Another interesting observation of our study was that the WT proSP-C BRICHOS domain, compared to the monomer mutant, has an ∼5-fold lower apparent affinity for immobilized Aβ42 fibrils. Nevertheless, both BRICHOS domain variants appear to be equally efficient in preventing Aβ42 fibril formation, and only at high protein concentrations is the monomer mutant slightly more efficient. This difference could be explained by the lower affinity of the proSP-C BRICHOS trimer conformation for fibrils, by bulk effects close to the fibrillar binding sites or a shift of the equilibrium between different quaternary structures in WT proSP-C BRICHOS preparations. However, our results strongly suggest that WT trimers bind to Aβ42 secondary nucleation competent aggregates and contribute to the observed inhibition of Aβ42 aggregation. Previously, molecular dynamics simulations suggested that only the proSP-C BRICHOS monomer can expose its potential substrate binding site while necessary conformational changes are blocked in the trimer.^16^ We speculate that the trimer might adopt a binding competent conformation independent of its monomerization but with weaker affinity for Aβ42 aggregates.

## Conclusion

Molecular structures of Aβ42 oligomers and interactions between molecular chaperones and small Aβ aggregates have been shown,^7,25,32^ but the abilities of these aggregates to promote secondary nucleation in relation to their size remained unknown. Our findings extend the knowledge of the smallest secondary nucleation potent Aβ42 aggregates and their interactions with a molecular chaperone domain. The approach presented here can be used to study structural properties of amyloid aggregates using the unique properties of different molecular chaperones.

## Supporting Information Available

Additional figures, a table with global fitting parameters, and Methods (PDF)

## Accession Codes

The human proSP-C BRICHOS domain corresponds to amino acid residues 59–197 of human proSP-C (UniProt KB P11686), and Aβ42 spans residues 671–713 of the human amyloid-β precursor protein (UniProt KB P05067).
