# Supplementary material for: Smallest Secondary Nucleation Competent Aβ Aggregates Probed by an ATP-Independent Molecular Chaperone Domain
Source: Biochemistry. 2021 Feb 23;60(9):678–88. doi: 10.1021/acs.biochem.1c00003 (PMC8028046; doi:10.1021/acs.biochem.1c00003)
Supplement: Supplementary file 1 — bi1c00003_si_001.pdf [file bi1c00003_si_001.pdf]

Supplementary Information

Smallest secondary nucleation competent Aβ aggregates probed by an ATP-independent molecular chaperone domain

Axel Leppert [†§], Ann Tiiman [¥], Nina Kronqvist [†§], Michael Landreh [‡], Axel Abelein [†§], Vladana Vukojević [¥*] and Jan Johansson[†§*]

[†] Department of Neurobiology, Care Sciences and Society, Division of Neurogeriatrics, Karolinska Institutet, 14183 Huddinge, Sweden

[¥] Department of Clinical Neuroscience, Center for Molecular Medicine, Karolinska Institutet, 17176 Stockholm, Sweden

[‡] Department of Microbiology, Tumour and Cell Biology, Karolinska Institutet, Biomedicum, Solnavägen 9, 171 65 Solna, Sweden

[§] Current address: Department of Biosciences and Nutrition, Karolinska Institutet, 14183 Huddinge, Sweden

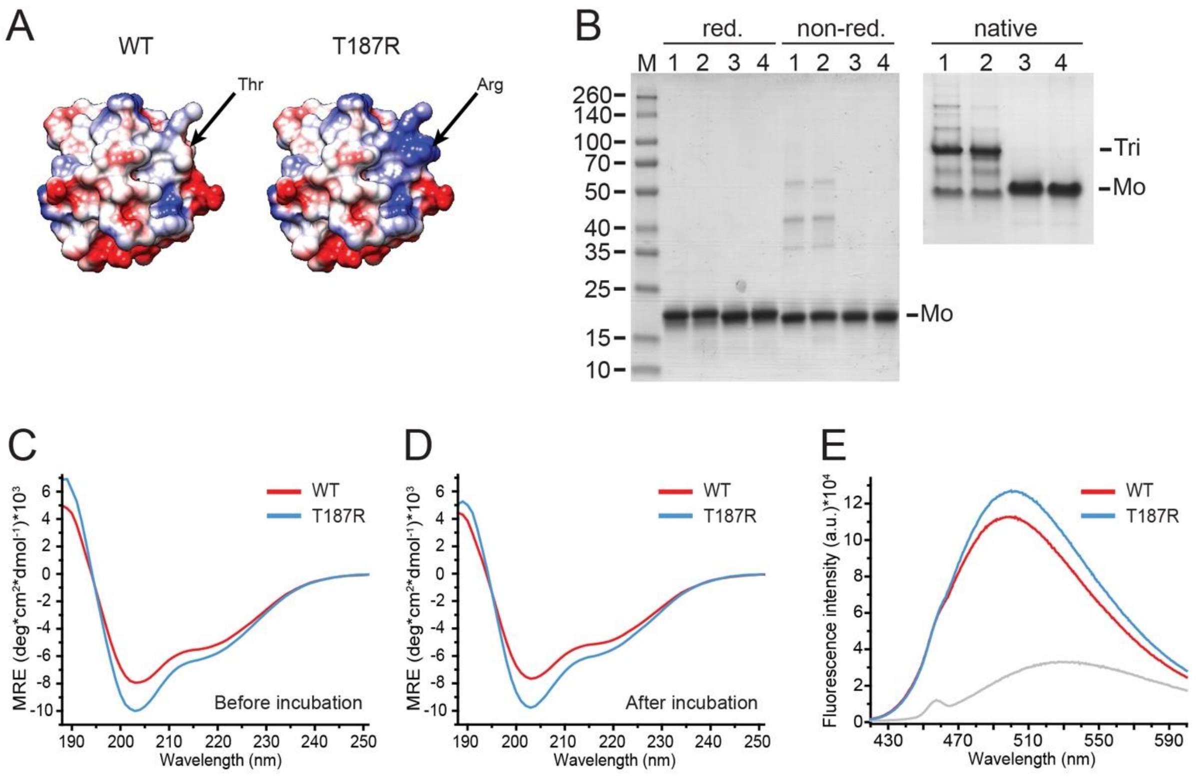

Figure S1. Characterization of proSP-C BRICHOS WT and proSP-C BRICHOS T187R. (A) proSP-C BRICHOS subunits with coulombic surface coloring from -5 kcal/mol*e (red) to 5 kcal/mol*e (blue). The arrows indicate the position of Thr187 and Arg187. (B) SDS-PAGE analysis under reducing and non-reducing conditions, and native PAGE. Lanes correspond to proSP-C BRICHOS WT before (1) and after (2) incubation, and proSP-C BRICHOS T187R before (3) and after (4) incubation. The apparent molecular weights were compared to a protein standard which is given to the left (lane M). (C) Far-UV CD spectra before and (D) after incubation. (E) Bis-ANS fluorescence spectra with (red and blue) or without (grey) proSP-C BRICHOS.

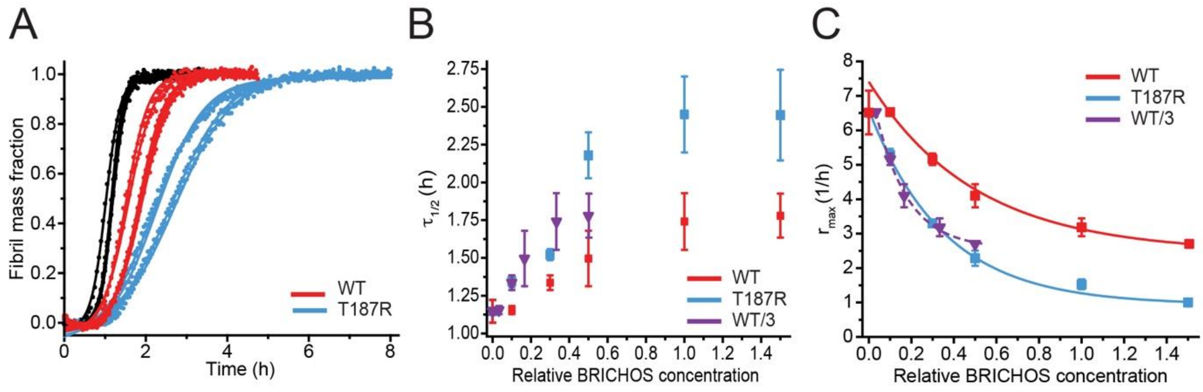

Figure S2. ProSP-C BRICHOS effects on Aβ42 aggregation. (A) Individual fits (solid lines) of normalized aggregation traces (circles) of 3 μM Aβ42 alone (black) and in the presence of equimolar WT proSP-C BRICHOS (red) or proSP-C BRICHOS T187R (blue). The traces correspond to 4 individual replicates each and the molar equivalents are based on monomeric protein solutions. Sigmoidal fitting parameters (B) $\tau_{1/2}$ and (C) r_{max} calculated from the aggregation of 3 μM Aβ42 with increasing proSP-C BRICHOS concentrations. Stoichiometrically corrected fitting parameters of proSP-C BRICHOS WT are shown as purple triangles. The lines are fits to the data using a mono-exponential decrease function. Data are presented as a mean ± s.d. of 4-5 technical replicates.

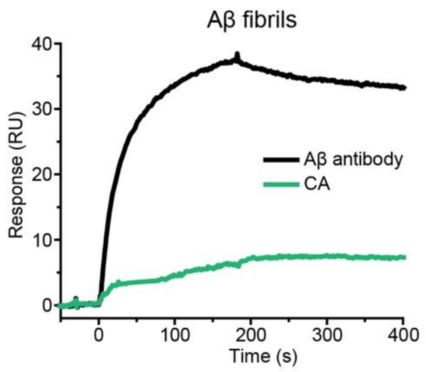

Figure S3. SPR sensorgrams of 0.1 μM Aβ antibody (6E10; black) and 50 μM carbonic anhydrase (green) binding to immobilized Aβ42 fibrils. Datasets were repeated two times with similar results.

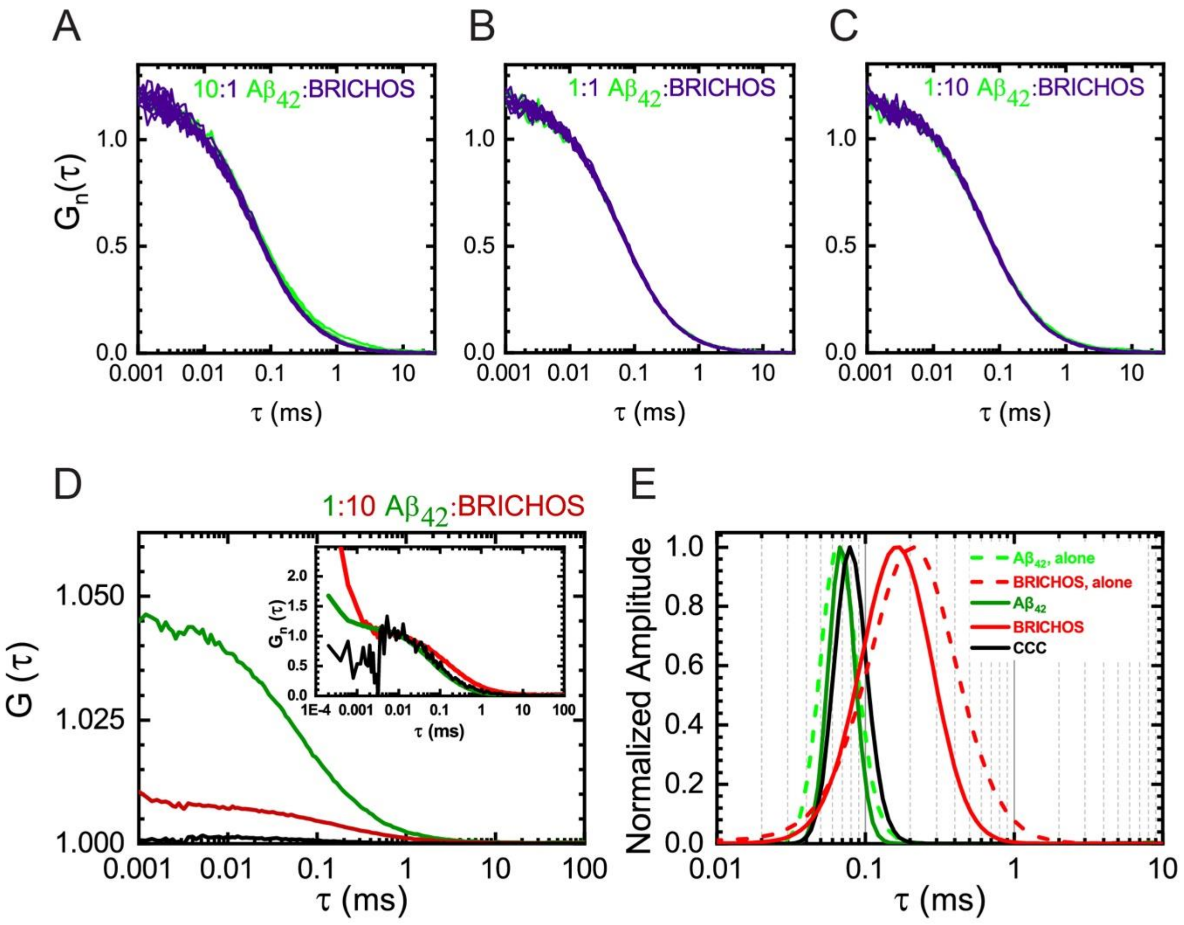

Figure S4. ProSP-C BRICHOS T187R and Aβ42 do not interact as monomers. (A-C) Normalized ACCs recorded in a 50 nM HiLyteFluor488-Aβ42 solution (light green) and with different amounts of unlabeled proSP-C BRICHOS T187R (lilac): (A) 5 nM, (B) 50 nM and (C) 500 nM. (D) ACCs recorded in a mixture of 50 nM HiLyteFluor488-Aβ42 (dark green) and 500 nM proSP-C BRICHOS T187R-Atto655 (red) and the corresponding CCC (black). *Inset*: ACCs normalized to the same amplitude. (E) MEMFCS analysis of the data shown in (D) (solid line curves colored as in (D)), and the distribution of diffusion times in solutions of HiLyteFluor488-Aβ42 and proSP-C BRICHOS T187R-Atto655 alone (dashed green and red curves, respectively).

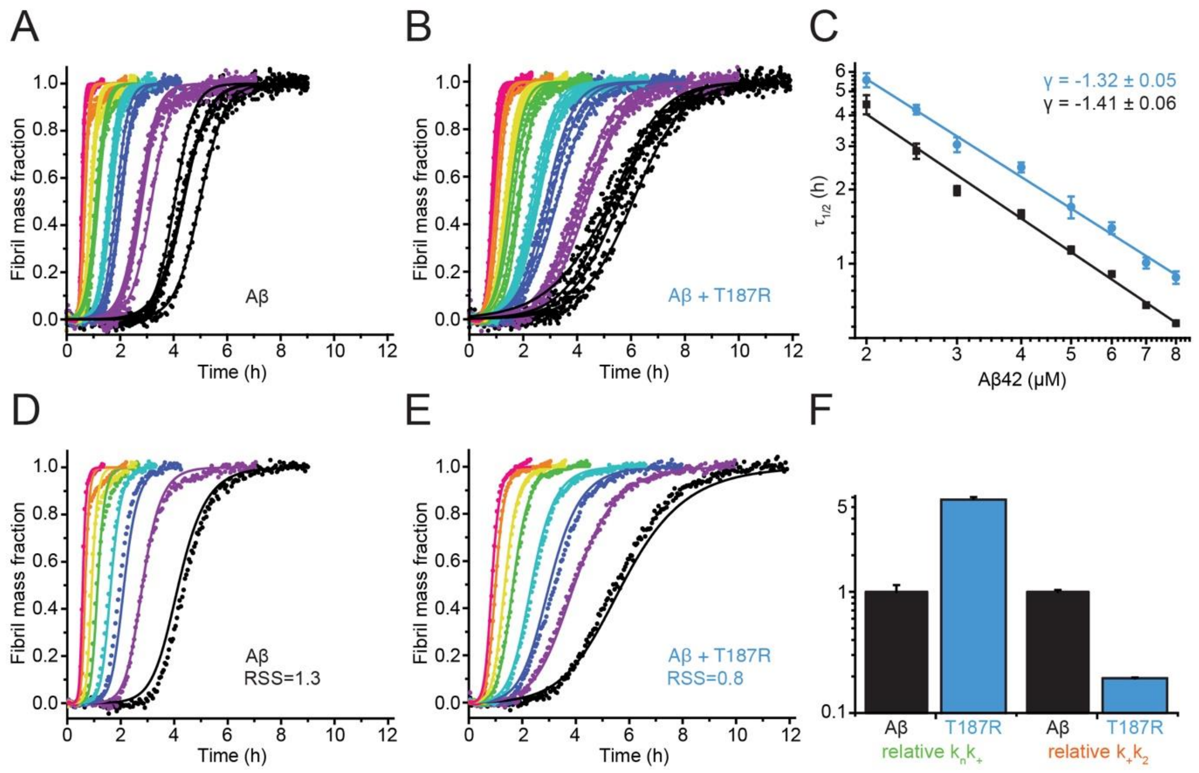

Figure S5. Kinetics of Aβ42 aggregation in the presence of constant proSP-C BRICHOS T187R concentrations. (A) and (B) Sigmoidal fits (solid lines) of Aβ42 aggregation traces (circles) in the absence and presence of 0.9 µM proSP-C BRICHOS T187R with different Aβ42 monomer concentrations: 2 (black), 2.5 (purple), 3 (blue), 4 (turquoise), 5 (green), 6 (yellow), 7 (orange) and 8 µM (red). (C) Dependence of the aggregation $\tau_{1/2}$ on the initial Aβ42 monomer peptide concentration in the absence (black) and presence (blue) of proSP-C BRICHOS T187R. Data are presented as mean ± s.d. of 4 technical replicates as shown in (A) and (B). The line is a fit to the data using a linear regression function to the log-values. (D) and (E) Global fits (solid lines) of the averaged Aβ42 aggregation traces (circles) from (A) and (B) where the combined rate constants $\sqrt{k_n k_+}$ and $\sqrt{k_+ k_2}$ are shared fitting parameters across all peptide concentrations. The quality of the fit was judged from their residual sum of squares (RSS) and the corresponding fitting parameters are shown in Supplementary Table 2. (F) Relative effects of 0.9 µM proSP-C BRICHOS T187R (blue) on the combined rate constants that were globally fitted across all Aβ42 concentrations. Errors bars correspond to the fitting error.

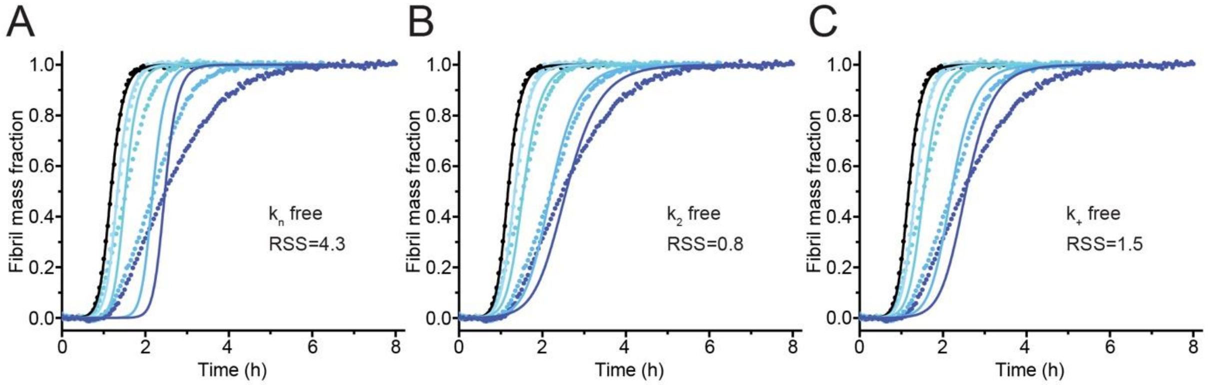

Figure S6. Kinetics of Aβ42 aggregation in the presence of varying proSP-C BRICHOS T187R concentrations. (A-C) Averaged aggregation traces of 3 μM Aβ42 (black) in the presence of 0.1, 0.3, 0.5, 1 molar equivalents of proSP-C BRICHOS T187R (light blue to blue) as shown in Figure 3A are globally fitted (solid lines) with a kinetic nucleation model across all proSP-C BRICHOS T187R concentrations. Only one rate constant is the free fitting parameter: (A) primary nucleation (k_n), (B) secondary nucleation (k_2), and (C) fibril-end elongation (k_+). The quality of the fit was judged from their residual sum of squares (RSS).

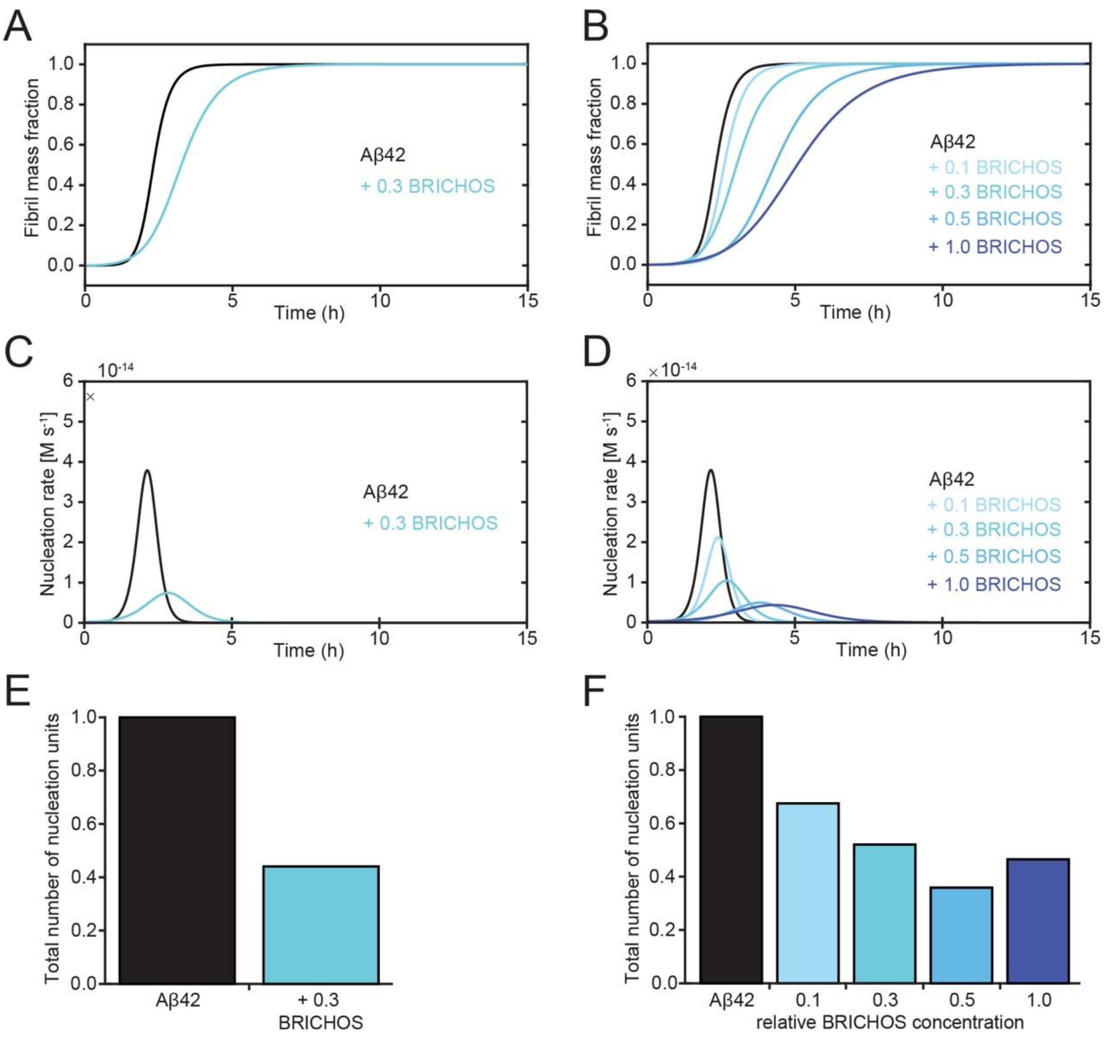

Figure S7. Time evolution of the nucleation rate and generation of Aβ42 nuclei in the absence and presence of proSP-C BRICHOS T187R. Calculated reaction profiles of Aβ42 in the absence (black) and presence (blue) of proSP-C BRICHOS T187R based on the rate constants determined by (A) global fitting of different Aβ42 concentrations with and without 0.3 molar equivalents proSP-C BRICHOS T187R (Figure S5D and S5E) and seeded aggregation experiments (Figure 3B); and (B) individual fitting of 3 μM Aβ42 with and without different molar equivalents proSP-C BRICHOS T187R and the elongation rates from seeded aggregation experiments (Figure 3B). (C) and (D) The corresponding time evolution of the nucleation rates calculated from the reaction profiles in (A) and (B). (E) and (F) Relative number of nucleation units formed in the absence (black) and presence of increasing molar equivalents of proSP-C BRICHOS T187R (light blue to blue).

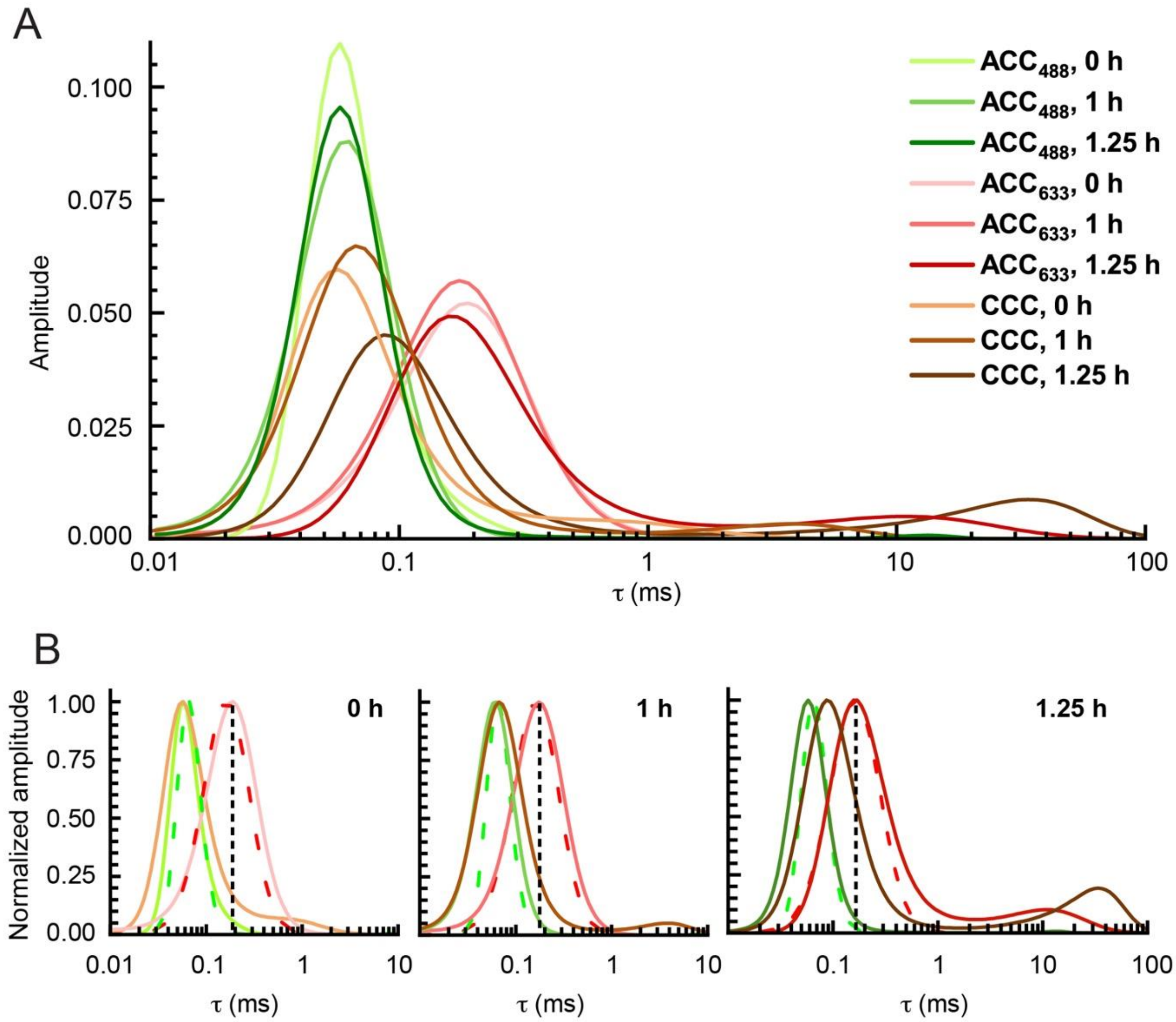

Figure S8. ProSP-C BRICHOS T187R binding to small soluble Aβ42 aggregates – analysis of cross-correlation curves (CCCs) in fluorescence cross-correlation spectroscopy (FCCS) measurements. (A) MEMFCS diffusion time distributions shown in Figure 4B (b_3 and bb_3) and Figure 5B (b_3), for HiLyteFluor488-Aβ42 (ACC_{488}; green), proSP-C BRICHOS T187R-Atto655 (ACC_{633}; red) and the CCCs of dually labelled Aβ42 aggregates in a complex with proSP-C BRICHOS T187R-Atto655 (brown) at time points t = 0 h (lightest), 1 h (darker) and 1.25 h (darkest). (B) Normalized MEMFCS diffusion time distributions shown in (A). ACC_{488} (green), CCC (brown) and ACC_{633} (red) at t = 0 h, 1 h and 1.25 h. Dashed line show the diffusion time distribution for proSP-C BRICHOS T187R-Atto655.

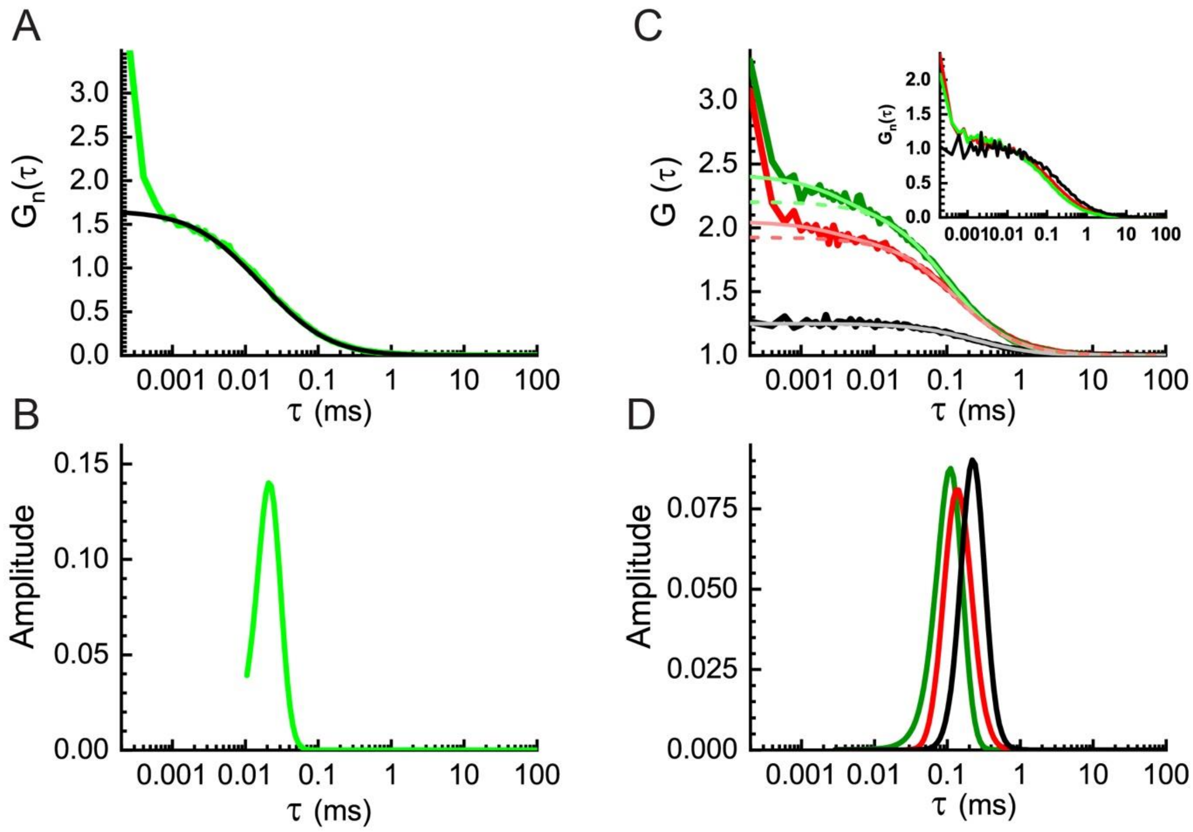

Figure S9. FCS instrument calibration. (A) Experimentally derived temporal autocorrelation curve for Rhodamine 6G (Rh6G) normalized to $G_n(\tau) = 1$ at lag time $\tau = 10$ μs (green) and the corresponding best-fit curve derived by MEMFCS (black). (B) Diffusion time distribution for Rh6G determined by MEMFCS analysis of data shown in (A), $\tau_{D,Rh6G} = (27 \pm 2)$ μs. (C) Experimentally derived temporal ACCs (dark green and red) and the CCC (black) for the IBA *In Vitro* FCCS Standard Probe 488/633 with corresponding best-fit curves. ACCs were fitted using a model for free 3D diffusion of a single species with a triplet term (light green and light red solid lines; the fraction of molecules in the triplet state was $T_{488} = 0.15$, $\tau_T = 3$ μs and $T_{633} = 0.11$, $\tau_T = 3$ μs) and without the triplet term (light green and light red dashed lines, to visualize the G_0 values). The CCC is fitted using the equation for free 3D diffusion of a single component without triplet term (light grey). *Inset:* The auto- and cross-correlation curves shown in (C) normalized to the same amplitude, $G(\tau) = 1$ at $\tau = 1\times10^{-5}$ s. (D) Diffusion time distribution determined by MEMFCS analysis of the data shown in (C), yielding $\tau_{D,488} = (100 \pm 15)$ μs, $\tau_{D,633} = (160 \pm 20)$ μs and $\tau_{D,CC} = (200 \pm 25)$ μs. The color code is the same as in (C).

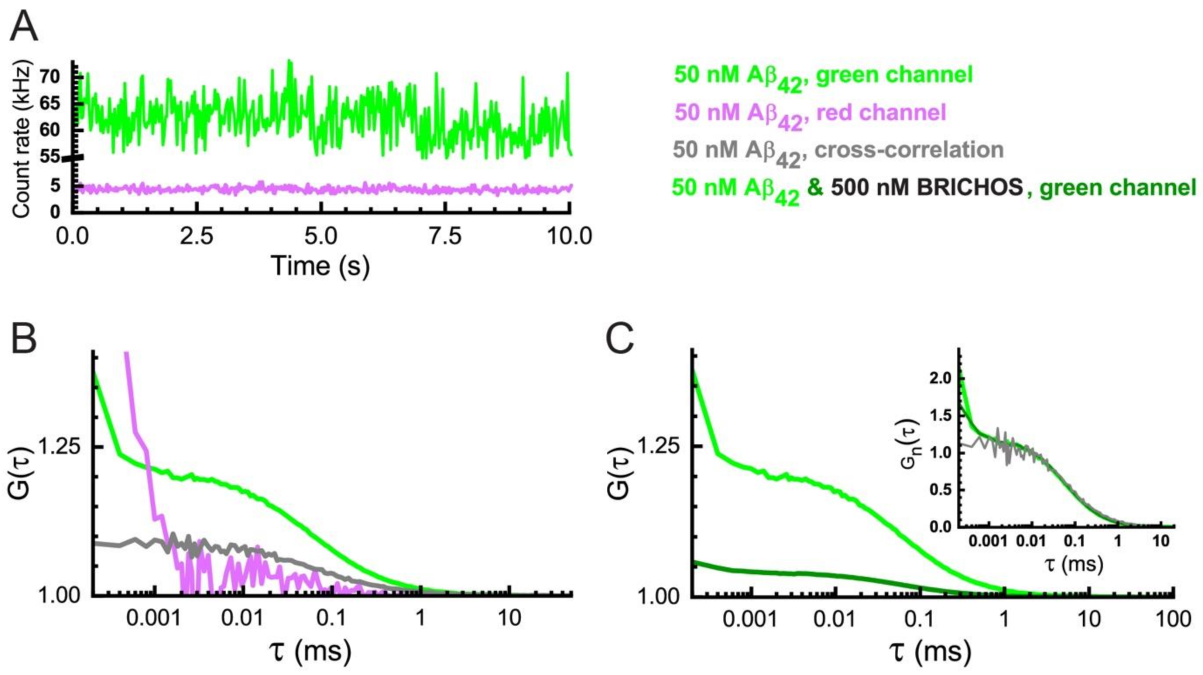

Figure S10. Effect of cross-talk between the channels on FCCS. (A) Fluorescence intensity fluctuations in the green channel (green) and signal bleed-through into the red channel (light red) upon 50 nM HiLyteFluor488-Aβ42 excitation at 488 nm. (B) ACCs (green and light red) and a CCC (light grey) derived by temporal auto- and cross-correlation analysis of the fluorescence intensity fluctuations shown in (A). (C) ACCs recorded in 50 nM HiLyteFluor488-Aβ42 alone (light green) and in the presence of 500 nM proSP-C BRICHOS T187R-Atto655 (dark green). Signal bleed-through from the red to the green channel increases the background in the green channel, which leads to a decrease in the amplitude of the ACC. *Inset*: ACCs normalized to the same amplitude, G(τ) = 1 at lag time τ = 10 μs, show that signal bleed-through from the red to the green channel does not alter the characteristic decay time of the ACC recorded in the green channel, as evident from the overlap between the corresponding ACCs (dark green *versus* green). The CCC (light grey), generated by temporal cross-correlation of the HiLyteFluor488-Aβ42 signal in the green channel with the bleed-through signal recorded in the red channel, overlaps with the ACC for HiLyteFluor488-Aβ42.

Table S1: Global fitting parameters of Aβ42 aggregation traces in the absence and presence of constant proSP-C BRICHOS T187R concentrations as shown in Figure S5D and S5E. Errors bars correspond to the fitting error.

	$\sqrt{k_n k_+}$ **in M^{-1}s^{-1}**	$\sqrt{k_+ k_2}$ **in M$^{-3/2}$s^{-1}**
**Aβ42**	3.57 ± 1.51	(1.61 ± 0.35)·10^5
**T187R**	8.73 ± 1.75	(0.74 ± 0.10)·10^5

Methods

Protein preparation and labeling of proSP-C BRICHOS T187R.

The recombinant human proSP-C BRICHOS domain corresponds to amino acid residues 59-197 of human proSP-C (UniProt KB accession number P11686). Wild type (WT) proSP-C BRICHOS was expressed and purified as previously described [1]. The monomer mutant was generated by substituting threonine at position 187 with arginine (T187R). After sequence verification, expression was carried out as described for proSP-C BRICHOS WT with minor changes: NaCl was omitted from all buffers and instead of an ion exchange column, monomeric proSP-C BRICHOS T187R protein was purified by size exclusion chromatography (SEC), using a Superdex 75 PG 26/600 column (GE Healthcare). Finally, protein concentrations were determined by measuring the absorbance at 280 nm and using a molar extinction coefficient of 9190 M^{-1} cm^{-1}. For analytical SEC a Superdex 75 column (GE Healthcare) was used and calibrated with several high and low molecular weight standards. Monomeric proSP-C BRICHOS T187R was fluorescently labeled using the primary amine reactive NHS ester derivate of Atto655 (Sigma Aldrich). Labeling was carried out in 20 mM NaPi, 0.2 mM EDTA buffer (pH 8) with a 4-fold molar excess of protein over dye. The mixture was incubated at room temperature for 30 min and the labeled protein was isolated from unbound dye by SEC using a Superdex 75 column (GE Healthcare). The degree of labeling was calculated by measuring the absorbance at 663 nm, using an extinction coefficient of $1.25*10^5$ M^{-1} cm^{-1} and a correction factor of 0.08.

Recombinant monomeric Met-Aβ(1-42) (hereafter referred to as Aβ42) spans the amino acids 671−713 of the human amyloid-β precursor protein (UniProt KB accession number P05067) and was expressed and purified from inclusion bodies as previously described[2]. Lyophilized Aβ42 aliquots were dissolved in 7 M guanidine hydrochloride (pH 8.5) and monomers purified

on a Superdex 30 PG 26/600 column (GE Healthcare) in 20 mM NaPi, 0.2 mM EDTA buffer (pH 8). Peptide concentrations were determined by measuring the absorbance A_{280}–A_{300} using a molar extinction coefficient of 1424 M^{-1} cm^{-1}. Monomeric HiLyteFluor488-Aβ42 (AnaSpec, USA) was diluted to the desired concentration with 20 mM NaPi, 0.2 mM EDTA, pH 8.

Preparation of sonicated Aβ42 fibrils.

Different concentrations of monomeric Aβ42 were incubated without agitation for 48 h at 37 °C in 20 mM NaPi, 0.2 mM EDTA buffer (pH 8). Fibrillated Aβ42 was sonicated on ice for a total time of 60 sec (2 sec on and 2 sec off, 30 % maximum amplitude) using a sonopuls ultrasonic homogenizer with a MS73 microtip (Bandelin). Sonicated fibrils were always kept on ice and directly used for immobilization on biosensor chips.

Bulk ThT fluorescence assays.

Aggregation kinetics were monitored in bulk solution by measuring total ThT fluorescence using a POLARstar Omega plate reader (BMG Labtech) with a 440 nm excitation filter and a 480 nm emission filter. All measurements were conducted at 37 °C, without agitation. Samples were prepared with 10 µM ThT in 20 mM NaPi, 0.2 mM EDTA buffer (pH 8) in black half-area 384-well polystyrene microplates with a transparent bottom (Corning). The reactant volume of each replicate was 20 µl. For kinetic experiments with constant Aβ42 concentrations, 3 µM Aβ42 was prepared in the absence and presence of varying molar ratios of both proSP-C BRICHOS variants. Kinetic experiments with a constant proSP-C BRICHOS T187R concentration (0.9 µM) were performed in presence of varying Aβ42 concentrations from 2-8 µM.

To determine the effects of different proSP-C BRICHOS T187R concentrations on the aggregation half time and maximum growth rate of Aβ42 fibril formation, fluorescence data were normalized and fitted to an empirical sigmoidal function[3]:

$$F = F_0 + A/(1 + \exp(r_{max}(\tau_{1/2} - t))) \quad (1)$$

where F_0 is the baseline value, A the amplitude, r_{max} the maximum growth rate, and $\tau_{1/2}$ the aggregation half time.

The aggregation half time $\tau_{1/2}$ of Aβ42 fibril formation is dependent on the initial monomer peptide concentration m(0) and can be expressed through a power law function[4]:

$$\tau_{1/2} \propto m(0)^{\gamma} \quad (2)$$

where γ is the scaling exponent, $\tau_{1/2}$ the aggregation half time and m(0) the monomer peptide concentration.

To investigate the effects of the proSP-C BRICHOS T187R domain on the microscopic rate constants in the Aβ42 aggregation process, data were fitted to a kinetic nucleation model that integrates rate laws for filamentous growth[5, 6]:

$$\frac{M(t)}{M(\infty)} = 1 - \left(\frac{B_+ + C_+}{B_+ + C_+ * \exp(\kappa t)} * \frac{B_- + C_+ * \exp(\kappa t)}{B_- + C_+}\right)^{\frac{k_\infty^2}{\kappa \tilde{k}_\infty}} * \exp(-k_\infty t) \quad (3)$$

where M(t) is the total fibril mass at time t and the coefficients $B_\pm$, $C_\pm$, k_∞, and $\tilde{k}_\infty$ are functions of λ and κ: $B_\pm = (k_\infty \pm \tilde{k}_\infty)/2/\kappa$; $C_\pm = \pm\lambda^2/2/\kappa^2$; $k_\infty = \sqrt{2\kappa^2/(n_2(n_2+1)) + 2\lambda^2/n_c}$; $\tilde{k}_\infty = \sqrt{k_\infty^2 - 4C_+C_-\kappa^2}$.

λ and κ are expressed by the microscopic rate constants for primary nucleation (k_n), fibril elongation (k_+), and secondary nucleation (k_2) with $\lambda = \sqrt{2 * k_+k_n * m(0)^{n_c}}$ and $\kappa =$

$\sqrt{2 * k_+ k_2 * m(0)^{n_2+1}}$, where n_c and n_2 are the reaction orders for primary and secondary nucleation, respectively.

Aβ42 seeds were prepared by incubating 80 µl of 3 µM monomeric Aβ42 in 20 mM NaPi, 0.2 mM EDTA buffer (pH 8) in 96-well polystyrene microplates with a transparent bottom (Corning) at 37 °C for 18 h. Formed fibrils were collected and sonicated in a water bath for 3 min. Aβ42 seed concentrations were calculated based on the initial Aβ42 monomer concentration. Seeding experiments were performed by adding 0.6 µM Aβ42 seeds to 3 µM monomeric Aβ42 in the absence and presence of different molar ratios of proSP-C BRICHOS T187R with 10 µM ThT in 20 mM NaPi, 0.2 mM EDTA buffer (pH 8). Fluorescence measurements were performed as previously described. To determine the elongation rate k_+ from the seeding experiments, the datapoints at the beginning of each curve where fitted with a linear equation and the slope plotted against the relative proSP-C BRICHOS T187R concentration.

To estimate the generation of nucleation units based on the previously calculated nucleation rates, the time evolution of the formation of new nuclei by monomers through secondary nucleation mechanisms was calculated. Calculations were based on the rate constants obtained from either the concerted aggregation kinetics of eight Aβ42 concentrations in the absence or presence of 30 % proSP-C BRICHOS T187R or from the individual fits derived from 3 µM Aβ42 in the absence and presence of varying concentrations of proSP-C BRICHOS T187R[5]:

$$r_n(t) = k_n m(t)^{n_c} + k_2 M(t) m(t)^{n_2} \tag{4}$$

where $r_n(t)$ is the nucleation rate.

Surface plasmon resonance (SPR).

SPR assays were performed in a BIAcore 3000 instrument (BIAcore AB). Aβ42 monomers or Aβ42 fibrils were individually immobilized by amine coupling onto flow-cells on different types of sensor chips (GE Healthcare). All immobilization experiments were performed with phosphate buffer (20 mM sodium phosphate, 0,2 mM EDTA, pH 8.0) as running buffer, a flow rate of 20 μL/min^{-1} and otherwise according to the manufacturer's instructions. Blank reference surfaces were prepared on flow-cell 3 on each sensor chip using the same coupling protocol only with no protein injected. Sonicated Aβ42 fibrils diluted in phosphate buffer to a concentration of 150 μM (based on monomeric subunits) were immobilized onto flow-cell 4 on a C1 sensor chip by 5 x 10 min manual injections to reach saturation and a final immobilization level of 480 resonance units (RU). Aβ42 monomers diluted in 10 mM sodium acetate buffer, pH 4.5 to a concentration of 2.5 μM were immobilized onto flow-cell 4 on a CM5 sensor chip using the amine-coupling immobilization wizard in the BIAcore 3000 control software, aiming for an immobilization level of 300 RU. After immobilization the flow-cells were stabilized over-night in HEPES-buffered saline without detergent (10 mM HEPES, 150 mM NaCl, 0.2 mM EDTA, pH 7.5) at a flow rate of 20 μL/min-1 to remove unspecifically bound protein.

Analytes for interaction studies were diluted in HEPES-buffered saline to 8 different concentrations between 0,4 μM – 50 μM for proSP-C BRICHOS WT, T187R and carbonic anhydrase from bovine erythrocytes (Gel Filtration Calibration Kit; GE Healthcare) or 100 nM for monoclonal antibody 6E10 (80300, BioLegend). The samples were individually injected in duplicates over the chip surfaces at 25 °C and at a flow rate of 30 μL/min^{-1}. All experiments were performed with HEPES-buffered saline as running buffer and 10-30 mM NaOH for the

regeneration of the chip surfaces. The response from the blank surface was subtracted from the immobilized surface response for each concentration of analyte.

Analysis of SPR data.

SPR sensorgrams were adjusted to zero and buffer spikes excluded. Dissociation of the analyte from the bound ligand is concentration independent. Since the change of the response signal for the dissociation phase of the three lowest protein concentrations was small, only sensorgrams obtained from BRICHOS samples ranging from 3.13 µM to 50 µM were included in the fit. The dissociation phase was fitted globally to a biexponential model [7]:

$$R(t) = R_1(xe^{-k_{d1}(t-t_1)} + (1-x)e^{-k_{d2}(t-t1)}) \qquad (5)$$

where the dissociation rate constants for the fast and slow phase k_{d1} and k_{d2}, respectively are global fit parameters, t_1 is the starting time of the dissociation phase, R_1 is the response signal at t_1 and x is allowed to vary between 0 and 1.

The association rate constants were calculated by analyzing the SPR data sets of BRICHOS concentrations ranging from 0.391 µM to 12.5 µM using the BIAevaluation software (Version 4.1). Sensorgrams were fitted to a heterogeneous ligand binding model using global kinetic fitting, with the dissociation rates determined by equation (6) set to constant and local adjustment of the parameter R_{max} (Figure S3C and D).

In order to validate the heterogeneous ligand fitting model, the association phase for all tested concentrations was fitted monoexponentially (data no shown) to:

$$R(t) = R_f + (R_0 - R_f)e^{-k_{obs}t} \qquad (6)$$

where R_f is the final response signal, R_0 the initial response signal and k_{obs} the observed rate constant.

The association rate is concentration dependent and linear regression analysis has been used to determine the fast (BRICHOS concentrations ranging from 0.391 µM to 3.13 µM) and slow (BRICHOS concentrations ranging from 3.13 µM to 50 µM) rate constants (data not shown):

$$k_{obs} = ck_a + k_d \qquad (7)$$

where c is the protein concentration, k_a is the association rate constant and k_d is the dissociation rate constant.

The apparent K_D values (Table 1) were calculated as ratio of the corresponding dissociation rate constants and association rate constants.

Fluorescence correlation and cross-correlation spectroscopy.

Fluorescence correlation spectroscopy (FCS) and fluorescence cross-correlation spectroscopy (FCCS) measurements were performed using the ConfoCor 2 system (Carl Zeiss, Jena, Germany) consisting of an inverted microscope (Axiovert 200 M) equipped with a C-Apochromat 40× NA = 1.2 water immersion UV-VIS-IR objective and avalanche photodiode detectors (SPCM-AQR-1X; PerkinElmer). HiLyteFluor488-Aβ42 fluorescence was excited using the 488 nm line of the Argon ion laser, whereas proSP-C BRICHOS T187R-Atto655 fluorescence was excited using the HeNe 633 nm laser. The emitted light was separated from the incident light by the HFT 488/633 main dichroic beam splitter. For FCCS, the emitted light was split using the secondary dichroic beam splitter NFT 635. HiLyteFluor488-Aβ42 fluorescence was transmitted to the detector through a band-pass filter BP 530-600, a long-pass filter LP 650 was used for proSP-C BRICHOS T187R-Atto655. For FCS measurements, the pinhole size in front of the detector was 70 µm for HiLyteFluor488 and 90 µm for Atto655. In FCCS experiments, the pinhole size was 90 µm for both channels.

For FCS/FCCS measurements, the reactants were dissolved in 20 mM NaPi, 0.2 mM EDTA, pH 8.0 and the reaction was allowed to proceed at T = 20 °C without agitation. Deionized water was used as a solvent throughout. For assessing monomer-monomer interactions, the HiLyteFluor488-Aβ42 peptide was diluted to a final concentration of 50 nM and proSP-C BRICHOS T187R binding to HiLyteFluor488-Aβ42 was assessed through changes in the characteristic decay time of the temporal autocorrelation curve (ACC) for HiLyteFluor488-Aβ42. In FCCS measurements, cross-correlation curves (CCCs) were analyzed to assess binding between HiLyteFluor488-Aβ42 and proSP-C BRICHOS T187R-Atto655. For monitoring proSP-C BRICHOS T187R interactions with aggregation intermediates of Aβ42, mixtures of unlabeled and labeled HiLyteFluor488-Aβ42 and proSP-C BRICHOS T187R were used. In particular, solutions containing 5, 10 or 20 µM of unlabeled Aβ42 with 100 nM HiLyteFluor488-Aβ42 and 384 nM of unlabeled proSP-C BRICHOS T187R with 100 nM of labeled proSP-C BRICHOS T187R-Atto655 were examined.

The reaction was followed over 12 hours. For each individual time point, fluorescence intensity fluctuations were recorded in 30 consecutive measurements, each measurement lasting 10 s, and an average ACC was calculated.

The Maximum Entropy Method for FCS (MEMFCS) [8] was used for bias-free fitting of the ACCs and CCCs, using the theoretical equation for polydisperse systems with *n* dissolved non-interacting components and one triplet state of the fluorophore used to label the peptide/protein monomers:

$$G(\tau) = 1 + \frac{1}{N} \cdot \left[1 + \frac{T}{1-T} \exp\left(-\frac{\tau}{\tau_T}\right)\right] \cdot \left(\sum_{i=1}^{n} \frac{f_i}{(1 + \frac{\tau}{\tau_{D_i}}) \sqrt{1 + \frac{w_{xy}^2}{w_z^2} \frac{\tau}{\tau_{D_i}}}} \right) \quad (8)$$

where N is the average number of molecules in the observation volume element (OVE); T the average equilibrium fraction of organic fluorophore molecules in the triplet state; τ_T the triplet correlation time; f_i the relative amplitude of the i-th component weighted by its brightness, and the sum of all weighted relative amplitudes is equal to 1, $\sum_{i=1}^{n} f_i$; τ_{Di} is the translational diffusion time of the i-th component; and w_{xy} and w_z are the $1/e^2$ lateral and axial radii of the confocal volume, respectively. The relative amplitude of the i-th component weighted by its brightness is $f_i = \frac{q_i^2 N_i}{\left(\sum_{i=1}^{n} q_i N_i\right)^2}$, where q_i is the ratio of the brightness of the i-th component to that of the brightness of a species chosen as a reference. Here, the monomer brightness measured at the beginning of the experiment was chosen as a reference. For CCCs analysis, the triplet term in eq. (8) was omitted since singlet-triplet transitions occur independently in different molecules and therefore do not give rise to the cross-correlation. We note also that the amplitude of the individual ACCs only depends on the overall abundance of the green- (N_{488} + $N_{488,633}$) and the red- (N_{633} + $N_{488,633}$) labeled molecules, whereas the amplitude of the CCC depends on the abundance of all species present, $N_{488,633}/(N_{488} + N_{488,633})(N_{633} + N_{488,633})$.

Instrument calibration was performed using freshly prepared standard solutions of Rhodamine 6G (Rh6G; Figure S9) and the IBA *In Vitro* FCCS Standard Probe 488/633 (IBA GmbH, Germany; Figure S9). Calibration was performed before the measurement series and checked afterwards to ascertain that signal quality did not significantly deteriorate over time. Rh6G diffusion time was determined to be $\tau_{D,Rh6G}$ = (27 ± 2) μs, and the diffusion time of the oligonucleotide cross-correlation standard was determined to be $\tau_{D,CCS}$ = (200 ± 25) μs (Figure S9). The ACCs reflected the expected difference in OVE_{488} and OVE_{633} size, which scale with the wavelength of the excitation light, as evident from the lower amplitude of the ACCs at lag time $\tau = 0$ ($G_{0,633}$ = 0.8 and $G_{0,488}$ = 1.3, Figure S9) and the longer diffusion time of the cross-correlation standard for the longer excitation wavelength ($\tau_{D,633}$ = (160 ± 20) μs *vs* $\tau_{D,488}$ = (100

± 15) µs). Data for Rh6G were used to determine the $1/e^2$ lateral radius of the Gaussian profile using the well-known relationship $\tau_D = \omega^2_{xy}/4D$ and $D_{Rh6G} = 4.14\times10^{-10}$ m^{2}s^{-1}, yielding $\omega_{xy,488} = 2.12\times10^{-7}$ m, and the aspect ratio for the OVE_{488}, $(\omega_z/\omega_{xy})_{488} = 3.65$. Subsequently, the diffusion coefficient of the cross-correlation standard was determined, $D_{CCS} = \tau_{D,Rh6G}\cdot D_{Rh6G}/\tau_{D,CCS} = (1.1 \pm 0.1)\times10^{-10}$ m^{2}s^{-1}. From this, the $1/e^2$ lateral radius of the red Gaussian profile, $\omega_{xy,633} = 2.68\times10^{-7}$ m, was derived based on the measured difference in the diffusion times $\tau_{D,633}/\tau_{D,488} \approx 1.6$. The aspect ratio for the OVE_{633}, $(\omega_z/\omega_{xy})_{633} = 2.9$, determined based on the measured difference in the amplitudes of the ACCs, $G_{0,633}/G_{0,488} = 1.6$, and the effective volume size $V_{eff,488} = \pi^{3/2}\cdot\omega^2_{xy,488}\cdot\omega_{z,488}$, was somewhat lower than the value determined independently in single-color calibration experiments ($(\omega_z/\omega_{xy})_{633} = 3.65$; data not shown), indicating that not all molecules in the cross-correlation standard are dually labeled.

The amplitude of the CCC relative to the amplitude of the ACC in the green channel, $G_{0,CCC}/G_{0,488} \approx 25$ %, which was somewhat lower than expected for a freshly synthesized oligonucleotide cross-correlation standard, $A_{CCC}/A_{488} \approx 35$ % at lag time $\tau = 10$ µs (https://www.iba-lifesciences.com/isotope/5/5-0000-504-Manual_invitro-FCCS.pdf), together with the fact that $\tau_{D,CC} > \tau_{D,633} > \tau_{D,488}$, indicated that the OVEs are eccentric, *i.e.* do not overlap (Figure S9) [9-11]. The CCC was fitted with the modified correlation function [9-11]:

$$G(\tau) = 1 + \frac{1}{c\cdot V_{eff,cc}\cdot N_A}\cdot\left(\frac{1}{1+\frac{4D_{CCS}\tau}{\omega^2_{xy,eff}}}\right)\cdot\left(\frac{1}{\sqrt{1+\frac{4D_{CCS}\tau}{\omega^2_{z,eff}}}}\right)\cdot exp\left(-\frac{d_x^2+d_y^2}{4D_{CCS}\tau+\omega^2_{xy,eff}}-\frac{d_z^2}{4D_{CCS}\tau+\omega^2_{z,eff}}\right), \quad (9)$$

where: c is molar concentration; $V_{eff,CC} = \pi^{3/2} \cdot \omega_{xy,488} \cdot \omega_{xy,633} \cdot \sqrt{\omega_{z,488} \cdot \omega_{z,633}}$ is the effective cross-correlation volume that is distinct from the geometrical average of $V_{eff,488}$ and $V_{eff,633}$ characteristic for concentric effective volumes [9-11]; N_A is Avogadro's number; $\omega^2_{xy,eff} = (\omega^2_{xy,488} + \omega^2_{xy,633})/2$ and $\omega^2_{z,eff} = (\omega^2_{z,488} + \omega^2_{z,633})/2$ define for the cross-correlation the 1/e^2 radii of the Gaussian profiles in the lateral and axial direction, respectively; and D_{CCS} is the diffusion coefficient of the cross-correlation standard, the lateral and axial displacement of the observation volumes due to chromatic aberrations of the objective and misalignment of emission volumes were determined to be $d_x = d_y = (80 \pm 20)$ nm and $d_z = (300 \pm 50)$ nm.

To find optimal FCCS measurement conditions, where the effects of signal bleed-through are minimal, we characterized the cross-talk following the procedure described by Bacia *et al.* [12]. To assess the signal bleed through from the green to the red channel, the apparent count rate ratio, f, was determined by measuring in 50 nM HiLyteFluor488-Aβ42 the average count rate in the red, CR_{633}, and the green, CR_{488}, channel upon excitation using the 488 nm line of the Ar ion laser, $f = CR_{633}/CR_{488} = 0.06 \pm 0.1$ (Figure S10). From the same measurements, the bleed-through ratio, κ, was derived using CPM as a proxy for HiLyteFluor488-Aβ42 brightness in the red and green channel, $\kappa = CPM_{633}/CPM_{488} = 0.02 \pm 0.01$ (Figure S10). Under such conditions, the error amplifying factor $a = 1/(1- \kappa f) = 1.0 \pm 0.1$, is smaller than the maximally permitted value $a = 2$ and the extent of cross-talk is acceptable to allow sufficiently accurate cross-talk correction [12]. Signal bleed-through from the red to the green channel was also observed (Figure S10). However, temporal autocorrelation analysis of the red signal into the green channel did neither give rise to an ACC, nor to a CCC, and the only effect on the green signal that was observed is that of an uncorrelated background, as reflected by the diminished amplitude of the ACC and its unaltered diffusion time (Figure S10).

Paired samples t-test, *i.e.* t-test for dependent means, performed using the free online T-Test Calculator (https://www.socscistatistics.com/tests/studentttest/default2.aspx), was used to compare paired values in a time series. For 5 µM Aβ42, the short decay time of the CCCs ($\tau_{D1,CCC}$ = 85 µs, SD = 24 µs) compared to the short decay time of the ACCs at 488 nm ($\tau_{D1,488}$ = 62 µs, SD = 4 µs), was significantly longer (t (77) = 6.24013, $p < 0.0001$); for 10 µM Aβ42, $\tau_{D1,CCC}$ = 74 µs, SD = 18 µs was significantly longer (t (76) = 4.53665, $p = 0.00021$) than $\tau_{D1,488}$ = 61 µs, SD = 3 µs; for 20 µM, $\tau_{D1,CCC}$ = 72 µs, SD = 21 µs was significantly longer (t (75) = 2.91575, $p = 0.004677$) than $\tau_{D1,488}$ = 62 µs, SD = 3 µs. The effect size, assessed using Cohen's delta (d), was large for 5 µM Aβ42 ($d = 0.96$), medium for 10 µM Aβ42 ($d = 0.72$) and medium, on the lower side, for 20 µM Aβ42 ($d = 0.26$). The short decay time of the ACC at 633 nm was always significantly longer than $\tau_{D1,CCC}$, $t > 17$ and $p < 0.0001$.

Circular Dichroism (CD) spectroscopy.

CD spectra in the far-UV region were recorded from 185 to 260 nm on a Chirascan CD spectrophotometer (Applied Photophysics) using a 1 mm quartz glass cuvette. The measurements were performed at 25 °C in 20 mM phosphate buffer (pH 8) containing 10 µM protein. Spectra represent the smoothed (over 3 points) average of 5 consecutive scans and were corrected for the corresponding buffer blank.

Bis-ANS fluorescence.

1 µM proSP-C BRICHOS was mixed with 2 µM 4,4′-Dianilino-1,1′-binaphthyl-5,5′-disulfonic acid (bis-ANS) and incubated for 10 min at 25 °C. Fluorescence emission spectra were measured with a Fluorolog-3 (Horiba) fluorescence spectrometer between 420 and 600 nm at an excitation wavelength of 395 nm.

Electrospray ionization mass spectrometry (ESI-MS).

Both proSP-C BRICHOS constructs were diluted to a final concentration of approximately 20 μM in 1 M ammonium acetate, pH 7.5. Mass spectra were acquired on a Micromass LCT ToF modified for analysis of intact protein complexes (MS Vision, The Netherlands) equipped with an offline nanospray source. ESI capillaries were purchased from Thermo. The capillary voltage was 1.5 kV, the cone voltage 1.5 kV, and the RF lens 1.5 kV. The pressure in the ion source was maintained at 9.0 mbar. Spectra were visualized using MassLynx 4.1 (Waters).

References

[1] Willander, H., Presto, J., Askarieh, G., Biverstal, H., Frohm, B., Knight, S. D., Johansson, J., and Linse, S. (2012) BRICHOS domains efficiently delay fibrillation of amyloid beta-peptide, *J Biol Chem 287*, 31608-31617.

[2] Walsh, D. M., Thulin, E., Minogue, A. M., Gustavsson, N., Pang, E., Teplow, D. B., and Linse, S. (2009) A facile method for expression and purification of the Alzheimer's disease-associated amyloid beta-peptide, *FEBS J 276*, 1266-1281.

[3] Abelein, A., Jarvet, J., Barth, A., Gräslund, A., and Danielsson, J. (2016) Ionic Strength Modulation of the Free Energy Landscape of Abeta40 Peptide Fibril Formation, *J Am Chem Soc 138*, 6893-6902.

[4] Cohen, S. I., Vendruscolo, M., Dobson, C. M., and Knowles, T. P. (2012) From macroscopic measurements to microscopic mechanisms of protein aggregation, *J Mol Biol 421*, 160-171.

[5] Cohen, S. I. A., Arosio, P., Presto, J., Kurudenkandy, F. R., Biverstal, H., Dolfe, L., Dunning, C., Yang, X., Frohm, B., Vendruscolo, M., Johansson, J., Dobson, C. M., Fisahn, A., Knowles, T. P. J., and Linse, S. (2015) A molecular chaperone breaks the catalytic cycle that generates toxic Abeta oligomers, *Nat Struct Mol Biol 22*, 207-213.

[6] Cohen, S. I., Linse, S., Luheshi, L. M., Hellstrand, E., White, D. A., Rajah, L., Otzen, D. E., Vendruscolo, M., Dobson, C. M., and Knowles, T. P. (2013) Proliferation of amyloid-beta42 aggregates occurs through a secondary nucleation mechanism, *Proc Natl Acad Sci U S A 110*, 9758-9763.

[7] Rahman, M. M., Zetterberg, H., Lendel, C., and Hard, T. (2015) Binding of Human Proteins to Amyloid-beta Protofibrils, *ACS Chem. Biol. 10*, 766-774.

[8] Sengupta, P., Garai, K., Balaji, J., Periasamy, N., and Maiti, S. (2003) Measuring size distribution in highly heterogeneous systems with fluorescence correlation spectroscopy, *Biophys J 84*, 1977-1984.

[9] Foo, Y. H., Naredi-Rainer, N., Lamb, D. C., Ahmed, S., and Wohland, T. (2012) Factors Affecting the Quantification of Biomolecular Interactions by Fluorescence Cross-Correlation Spectroscopy, *Biophys. J. 102*, 1174-1183.

[10] Weidemann, T., and Schwille, P. (2013) Dual-Color Fluorescence Cross-Correlation Spectroscopy with Continuous Laser Excitation in a Confocal Setup, In *Fluorescence Fluctuation Spectroscopy* (Tetin, S. Y., Ed.), pp 43-70, Elsevier Academic Press Inc, San Diego.

[11] Weidemann, T., Wachsmuth, M., Tewes, M., Rippe, K., and Langowski, J. (2002) Analysis of ligand binding by two-colour fluorescence cross-correlation spectroscopy, *Single Mol. 3*, 49-61.

[12] Bacia, K., Petrášek, Z., and Schwille, P. (2012) Correcting for spectral cross-talk in dual-color fluorescence cross-correlation spectroscopy, *Chemphyschem 13*, 1221-1231.
